# Plasma-Assisted and Green Routes to Silicon and Carbon Quantum Dots: A Critical Comparison of Synthesis–Structure–Property Relationships

**DOI:** 10.3390/nano16191273

**Published:** 2026-10-08

**Authors:** Sagi Orazbayev, Almasbek Utegenov, Assan Abdirakhmanov, Sultan Ussenkhan, Yerassyl Yerlanuly, Didar Batryshev, Moldir Nurusheva, Maratbek Gabdullin, Laifa Boufendi, Holger Vach, Tlekkabul Ramazanov

**Affiliations:** 1 Institute of Applied Sciences and Information Technologies, 280 Bayzakov Str., Almaty 050040, Kazakhstan; 2 NNLOT, Al-Farabi Kazakh National University, 71 Al-Farabi Ave., Almaty 050040, Kazakhstan; 3 Atyrau Oil and Gas University, 45a Baymukhanov Str., Atyrau 050027, Kazakhstan; 4 Plasma-Surface Interaction Chemistry (ChIPS) Laboratory, University of Mons, 20 Place du Parc, 7000 Mons, Belgium; 5 Kazakh-British Technical University, 59 Tole bi Str., Almaty 050000, Kazakhstan; 6 LPICM, CNRS, École Polytechnique, Institut Polytechnique de Paris, 91128 Palaiseau, France

**Keywords:** silicon quantum dots, carbon quantum dots, plasma synthesis, green synthesis, photoluminescence, quantum confinement, surface passivation, heteroatom doping, bioimaging

## Abstract

Silicon quantum dots (SiQDs) and carbon quantum dots (CQDs) are often discussed as related heavy-metal-free luminescent nanomaterials, although the mechanisms governing their formation and emission differ fundamentally. Rather than combining two parallel bodies of literature, this review uses SiQDs and CQDs as contrasting model systems to determine which synthesis–structure–property relationships are transferable and which remain material-specific. We critically compare nonthermal gas-phase plasma, plasma–liquid, and solution-phase synthesis, including green routes, with respect to precursor activation, nucleation and growth, crystallinity, surface chemistry, purification, photoluminescence, reproducibility, and scalability. Low-pressure continuous-flow radio-frequency capacitively coupled plasma (RF-CCP) is treated as the reference plasma architecture for free-standing Si nanocrystals, while atmospheric-pressure and plasma–liquid approaches are discussed according to their distinct product forms, formation mechanisms, and technological maturity. For crystalline SiQDs, quantum confinement provides a physically defined framework for interpreting size-dependent optical behavior, although oxidation, ligands, and interfacial defects remain critical. In CQDs, emission may arise from carbon-core states, surface or defect states, and molecular fluorophores, making structural assignment and fraction-resolved characterization essential. Across both material families, reported record photoluminescence efficiencies are less informative for technological translation than reproducibility, purified product yield, long-term stability, and application-specific performance. Hybrid plasma–liquid processing, operando diagnostics, standardized optical characterization, and data-guided process optimization are identified as promising directions toward scalable and reproducible quantum-dot manufacturing.

## 1. Introduction

The discovery of visible photoluminescence from porous silicon by Canham in 1990 is widely regarded as a foundational result in silicon photonics, demonstrating that quantum confinement could transform the indirect-bandgap semiconductor into an efficient light emitter [1]. This breakthrough catalyzed intensive research into silicon nanostructures, culminating in the development of colloidal silicon quantum dots (SiQDs) with tunable emission across the visible and near-infrared spectrum. In parallel, the serendipitous observation of bright fluorescence from carbon nanoparticles during electrophoretic purification of single-walled carbon nanotubes by Xu et al. in 2004 [2] inaugurated a new frontier in carbon-based nanophotonics, leading to the emergence of carbon quantum dots (CQDs) as a distinct class of luminescent nanomaterials [3].

SiQDs and CQDs share several practical advantages over conventional Cd- and Pb-containing quantum dots, including elemental abundance, adaptable surface chemistry, and access to visible or near-infrared photoluminescence. Halide perovskite quantum dots represent another important benchmark among high-brightness luminescent nanocrystals. Optimized perovskite compositions can achieve photoluminescence quantum yields approaching unity while maintaining narrow emission bandwidths, making them highly attractive for optoelectronic applications. However, their chemical, environmental, and operational stability remains a major challenge, while lead-containing compositions raise additional concerns related to toxicity and end-of-life disposal. These trade-offs highlight SiQDs and CQDs as complementary heavy-metal-free platforms whose advantages should be assessed in comparison with established high-performance nanophosphors rather than in isolation [4,5].

These similarities, however, can make an important distinction. SiQDs are semiconductor nanocrystals for which the dimensions and surface state of a defined Si core are central to the optical response, whereas CQDs comprise a structurally heterogeneous family in which carbon domains, surface states, defects, polymer-like structures, and molecular fluorophores may all contribute to emission. Consequently, the same descriptors—particle size, photoluminescence quantum yield (PLQY), or “surface passivation”—do not necessarily have equivalent physical meaning for the two material classes. The appeal of these nanomaterials may stem from several converging factors: (i) intrinsically low toxicity, with silicon and carbon being biocompatible elements abundant in biological systems; (ii) tunable photoluminescence spanning ultraviolet to near-infrared wavelengths through size control, surface engineering, and heteroatom doping; (iii) large Stokes shifts that minimize self-absorption and enable efficient light conversion; (iv) excellent photostability under ambient conditions; and (v) compatibility with scalable, cost-effective synthesis routes [6,7,8].

Among the methods used to prepare SiQDs and CQDs, plasma-based and solution-based routes have received particular attention. Plasma-assisted methods can employ either thermal or nonthermal plasmas; because thermal plasmas approach local thermodynamic equilibrium and typically favor larger, less controllable particles, most work on SiQDs and CQDs has instead relied on nonthermal plasmas, in which energetic electrons coexist with ions, radicals, excited species, and neutral molecules, to dissociate molecular precursors and initiate nanoparticle nucleation and growth under strongly non-equilibrium conditions. Nonthermal or cold plasmas are nonequilibrium systems in which the bulk gas temperature can remain close to room temperature despite the presence of highly energetic electrons. Compared with conventional solution-based and thermal gas-phase processes, nonthermal plasmas offer several distinctive advantages for nanomaterial synthesis. First, owing to their low mass, electrons are readily accelerated by electric fields and can acquire sufficiently high energies to drive excitation, ionization, and dissociation processes. Their interaction with growing nanoparticles generally results in the accumulation of negative charge. The resulting electrostatic repulsion can suppress particle coagulation and agglomeration, thereby facilitating the formation of very small nanoparticles with relatively narrow size distributions. Second, because electrons are considerably more mobile than ions, plasma-facing reactor surfaces typically acquire a negative potential (if they are not grounded), and plasma sheaths are formed near the walls. The associated electric fields can hinder the transport of negatively charged nanoparticles toward reactor boundaries, enhance their confinement within the plasma, and thereby increase their residence time and, under suitable conditions, the particle yield. Finally, electron-ion recombination, ion bombardment, and surface chemical reactions can release substantial amounts of energy directly at the nanoparticle surface. This localized energy transfer may raise the nanoparticle temperature significantly above that of the surrounding gas and promote crystallization even under comparatively low bulk-gas-temperature conditions [9,10].

These charging, confinement, and localized heating effects are harnessed in practice through a variety of reactor configurations developed for the synthesis of SiQDs and CQDs, including low-temperature capacitively coupled plasmas (CCPs), inductively coupled plasmas (ICPs), microwave discharges, atmospheric-pressure microplasmas, plasma-enhanced chemical vapor deposition (PECVD) and physical vapor deposition (PVD) systems, and plasma–liquid configurations. Their operating conditions can be adjusted over a broad range by varying the applied power or pulse characteristics, gas composition, pressure, precursor concentration, gas-flow rate, and particle residence time. This flexibility enables control over particle-size distributions, crystallinity, nucleation and growth kinetics, as well as surface chemistry and passivation, allowing the properties of the resulting nanomaterials to be tailored to specific applications [11,12,13,14,15]. The final structural and optical characteristics of SiQDs and CQDs are governed by the interplay among the electron energy distribution, precursor dissociation and radical-generation rates, particle charging and electrostatic confinement, plasma-surface interactions, and the transport of reactive species within the reactor [11,12,13,14,15].

Plasma-based approaches have been widely investigated for the synthesis of SiQDs and, more recently, carbon-based quantum dots because they allow nanoparticle formation and surface modification to be controlled through the discharge conditions [16,17,18,19,20,21,22,23]. In low-pressure silane plasmas, Si nanoparticles can be formed with hydride-rich surfaces, while changes in plasma chemistry, power, and molecular chemical species residence time strongly affect nucleation, growth, and the resulting size distribution [19,20,21,22,23]. Surface termination is particularly important for the optical properties of SiQDs, since hydrogen termination and subsequent oxidation or chemical passivation can reduce nonradiative recombination associated with surface defects [16,22,23,24]. For example, controlled formation of a SiO_2_ shell has produced Si/SiO_2_ core–shell quantum dots with photoluminescence quantum yields above 40% [24]. Recent plasma-based processing strategies have also produced SiQDs with internal quantum yields approaching unity, demonstrating that the intrinsic radiative efficiency can be very high when surface-related losses are sufficiently suppressed [25]; near-unity internal quantum efficiency has also been reported for SiQDs prepared by non-plasma techniques [26]. In parallel, plasma–liquid systems provide an alternative route for preparing silicon- and carbon-based nanostructures, including N-doped graphene quantum dots, and broaden the range of precursors and surface functionalities accessible through plasma-assisted synthesis [17,18].

In contrast to plasma-assisted techniques, green and solution-based approaches have been increasingly explored for the preparation of SiQDs and CQDs, particularly when low-cost precursors, relatively simple processing, and reduced use of hazardous chemicals are desired [8,27,28]. In the case of CQDs, hydrothermal and solvothermal treatment of biomass-derived feedstocks, including plant materials, agricultural residues, and food-processing waste, can induce dehydration, condensation, aromatization, and carbonization reactions that gradually produce nanoscale carbon domains [8,27,28]. The composition of the starting biomass also influences the surface chemistry of the resulting CQDs, since oxygen-, nitrogen-, or sulfur-containing species present in the precursor may contribute to surface functionalization or heteroatom incorporation during carbonization [8,27,28].

Microwave-assisted synthesis offers a faster alternative to conventional hydrothermal processing by providing rapid volumetric heating of the reaction mixture and shortening the time required for carbonization and nucleation [29,30]. The resulting optical properties, however, remain strongly dependent on precursor composition, reaction temperature, and the extent of carbonization, rather than on the heating method alone [29,30]. Laser ablation in liquids represents a different, predominantly physical route in which a pulsed laser interacts with a solid Si or carbon target immersed in a liquid, producing a transient plasma plume from which nanoscale species are generated through ablation, cooling, fragmentation, and condensation processes [31,32]. The surrounding liquid is not merely a passive medium; its composition can affect particle cooling, oxidation, stabilization, and ultimately the size and surface state of the resulting nanostructures [31,32].

Electrochemical methods provide another liquid-phase route, particularly for luminescent Si nanocrystals, where electrochemical etching of silicon followed by controlled chemical treatment can modify particle size, surface termination, and photoluminescence behavior [33]. Taken together, these approaches extend the range of accessible precursors and surface chemistries beyond plasma-based synthesis and offer considerable flexibility in tailoring quantum-dot properties. At the same time, their actual environmental benefit and scalability depend on the complete processing chain, including solvent choice, reagent consumption, energy demand, purification, and post-synthesis surface treatment.

Top-down chemical oxidation typically relies on concentrated acids and strong oxidizing agents and therefore requires extensive neutralization, purification, and separation to remove residual reagents and reaction byproducts. Nevertheless, acid oxidation followed by hydrothermal heteroatom doping has been successfully used to prepare large-scale N-, S-, and Se-doped CQDs with tunable photoluminescence, improved quantum yields, and longer fluorescence lifetimes than their undoped counterparts [34,35].

Irrespective of the synthesis route, careful control of precursor composition, reaction temperature, treatment time, and degree of carbonization is essential. Insufficient conversion may leave unreacted molecular species, whereas excessive carbonization can promote the formation of larger carbonaceous aggregates, soot-like particles, or polycyclic aromatic byproducts. These species complicate purification, interfere with the interpretation of photoluminescence mechanisms, and may reduce the isolated yield of CQDs [34,36].

Bottom-up methods, including hydrothermal and solvothermal treatments, microwave-assisted synthesis, pyrolysis, and thermal decomposition, generally offer considerable flexibility for tuning CQD composition, heteroatom incorporation, surface functionality, and particle-size distribution. However, they do not invariably offer better size control than top-down fragmentation methods, because the final properties depend strongly on precursor chemistry, reaction conditions, and subsequent purification or size fractionation [34,37]. Moreover, bottom-up synthesis is not restricted to high-purity molecular precursors. Renewable biomass, plant-derived materials, and food-processing or agricultural waste can also serve as effective carbon sources. However, variations in their composition may affect product uniformity and batch-to-batch reproducibility.

The optical response of SiQDs is strongly governed by the interplay between quantum confinement and surface-related electronic states. In crystalline SiQDs with dimensions below approximately 10 nm, spatial confinement of charge carriers increases the effective optical bandgap and relaxes, to some extent, the momentum constraints that limit radiative recombination in bulk silicon. Emission associated predominantly with the Si core is therefore commonly characterized by relatively long photoluminescence lifetimes, ranging from several to hundreds of microseconds [38]. Size-selected Si nanocrystals produced by gas-phase plasma from vaporized cyclohexasilane supplied as a liquid molecular precursor showed quasi-direct recombination behavior together with ensemble PLQYs approaching 70% [39], while optimized hydrogen-silsesquioxane-derived SiQDs have exhibited red emission with PLQYs in the range of approximately 60–80% [40]. These results indicate that highly efficient radiative recombination can be achieved in appropriately prepared and passivated Si nanocrystals, but they should not be taken as evidence for a universal indirect-to-direct bandgap transition in all sub-10 nm SiQDs. In practice, surface oxidation, ligand termination, dangling bonds, and interfacial defects introduce additional electronic states and competing recombination pathways, which can markedly influence the emission energy, intensity, lifetime, and photochemical stability of SiQDs [41,42].

The origin of photoluminescence in CQDs is heterogeneous, and no single mechanism adequately accounts for all reported materials. Depending on the precursor chemistry, synthesis conditions, degree of carbonization, and purification procedure, emission may originate from quantum-confined transitions within π-conjugated carbon domains, recombination through surface or defect states, and molecular fluorophores associated with or embedded in the carbonaceous matrix [3,43,44]. Crosslink-enhanced emissions may also contribute, particularly to polymer-rich carbon dots. Excitation-dependent photoluminescence often reflects the coexistence of several emissive centers rather than a single size-dependent electronic transition. Heteroatom doping with nitrogen, sulfur, phosphorus, boron, or combinations of these elements can modify the electronic structure, surface-state distribution, and balance between radiative and nonradiative recombination. Collectively, doped and co-doped CQDs with emission spanning the blue-to-red spectral range have been demonstrated [45,46,47,48]. Nevertheless, the emission color is determined not only by the dopant identity but also by the extent of π conjugation, the degree of carbonization, the core structure, and the surface functional groups.

Several reviews have separately summarized the synthesis, photophysics, and applications of SiQDs or CQDs. The purpose of the present review is therefore not simply to place two established bodies of literature side by side. Instead, SiQDs and CQDs are used as contrasting model systems to determine which synthesis–structure–property relationships are transferable across heavy-metal-free quantum dots, and which remain material-specific. This comparison is informative precisely because the two families can exhibit superficially similar optical outputs while relying on fundamentally different emissive structures: quantum-confined semiconductor cores modified by interfaces and surface chemistry in crystalline SiQDs, versus a potentially mixed population of carbon domains, defect/surface states, polymer-like structures, and molecular fluorophores in CQDs. The same framework is then applied across plasma-assisted, plasma–liquid, and solution-phase synthesis: precursor activation and nucleation, growth and structural definition, surface chemistry, purification, PLQY and lifetime, reproducibility, mass and energy efficiency, and application-specific performance. This approach exposes common reporting gaps while preventing inappropriate direct comparison of quantities that have different physical meanings. Applications are included only when material-level properties can be connected to device, sensing, environmental, or biological performance. The resulting review therefore provides a cross-material methodology for experimental design and standardized reporting rather than a catalogue of two quantum-dot bodies of literature.

## 2. Classification and Structural Architecture of Si/C Quantum Dots

Silicon and carbon-based quantum dots represent structurally diverse classes of nanomaterials that differ in their core composition, bonding configuration, surface chemistry, and optical response [3,6,7,36,37]. Particle size is an important factor, particularly when quantum-confinement effects become significant, but it is only one of several parameters that govern their electronic and photophysical behavior. Crystallinity, surface termination, interfacial bonding, heteroatom incorporation, and defect-related states can alter the electronic structure and introduce additional radiative or nonradiative recombination pathways [35,41,42,43,44,47,48]. As a result, quantum dots of similar dimensions may exhibit markedly different absorption and photoluminescence characteristics depending on their internal structure and surface environment.

**Figure 1:**
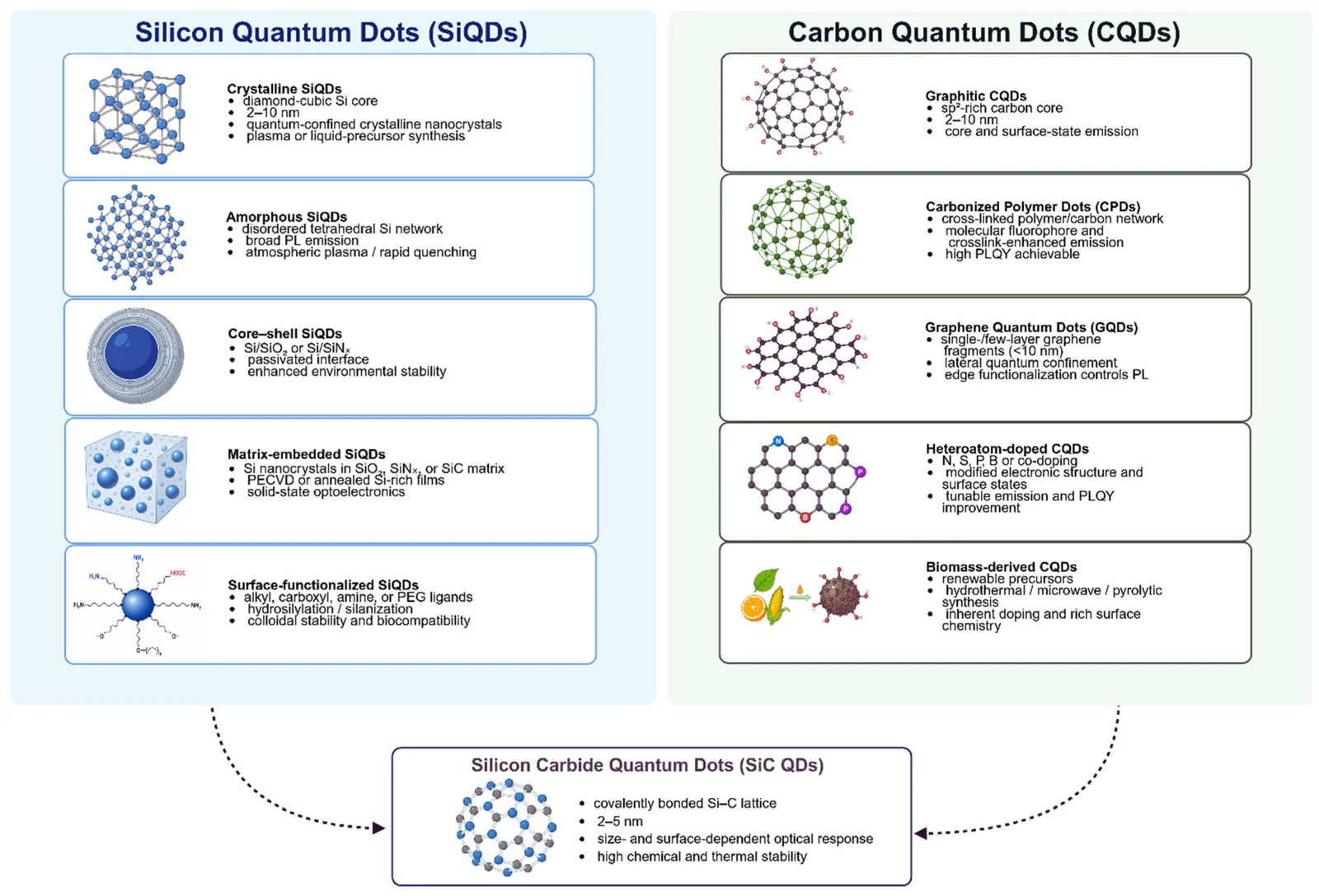
Structural classification and representative architectures of silicon quantum dots (SiQDs), carbon quantum dots (CQDs), and silicon carbide quantum dots (SiC QDs).

Figure 1 summarizes the classification adopted in this review. Silicon quantum dots are grouped into crystalline, amorphous or partially crystalline, core–shell, matrix-embedded, and surface-functionalized structures. Carbon-based dots are divided into graphitic carbon dots, carbonized polymer dots, graphene quantum dots, and heteroatom-modified systems. Biomass-derived CQDs are treated as a precursor-defined category because they may adopt more than one internal structure. Silicon carbide quantum dots are discussed separately as a hybrid Si-C family.

The structural features used to classify SiQDs and CQDs can overlap within the same nanomaterial. For example, a crystalline SiQD may simultaneously contain a partially oxidized surface and organic ligands, while a biomass-derived CQD may consist of a carbonized core or polymer-like framework decorated with nitrogen- or sulfur-containing functional groups. The proposed classification should therefore be viewed as a practical framework based on the predominant structural or compositional characteristics of each material, rather than as a set of strictly separated categories (Table 1).

### 2.1. Silicon Quantum Dots

#### 2.1.1. Crystalline SiQDs

Crystalline SiQDs contain partially diamond–cubic silicon domains with diameters commonly below approximately 10 nm. There are other crystalline structures that have been observed such as hcp, bc8 and fcc [66]. At these dimensions, quantum confinement modifies the electronic structure relative to bulk silicon, increases the effective optical bandgap, and partially relaxes the momentum-selection restrictions associated with indirect radiative recombination. Their emission energy and efficiency, however, are not determined by size alone. Crystallinity, internal strain, surface oxidation, ligand coverage, and interfacial defects all influence the balance between radiative and nonradiative pathways [39,40,49,50]. Plasma synthesis provides a direct route to free-standing Si nanocrystals. Energetic electrons dissociate silicon-containing precursors, after which nucleation, particle growth, crystallization, and surface termination occur within or downstream of the discharge. Atmospheric-pressure plasma studies have shown that precursor concentration, gas composition, plasma power, and particle heating can strongly affect nanocrystal size and crystallinity [49,50]. Alessi et al. obtained amorphous and crystalline SiQDs with mean diameters of approximately 1.5–5.5 nm and optical bandgaps of 2.3–2.5 eV [50]. Liquid silicon precursors can also be coupled with plasma processing. Pringle et al. prepared Si nanocrystals from cyclohexasilane and subsequently applied oxygen-protected size separation. The resulting fractions exhibited quasi-direct-like recombination and PLQYs approaching 70% [39]. These results also showed that efficient core emission depends on rigorous control of surface oxidation and particle-size distribution. Separately, thermally processed hydrogen-silsesquioxane-derived SiQDs exhibited red photoluminescence with PLQYs of approximately 60–80%, demonstrating that high efficiencies can be achieved through carefully controlled precursor conversion and surface passivation [40]. The crystalline structure of SiQDs is normally established using a combination of high-resolution transmission electron microscopy (HRTEM), selected-area electron diffraction, and X-ray diffraction. Resolved lattice fringes and diffraction features consistent with diamond–cubic silicon support the assignment of crystalline SiQDs. However, the absence of clearly resolved lattice fringes in extremely small particles does not necessarily prove that they are fully amorphous, because surface reconstruction and structural disorder may occupy a substantial fraction of the nanocrystal volume.

#### 2.1.2. Amorphous and Partially Crystalline SiQDs

Amorphous SiQDs lack the long-range translational order of diamond–cubic silicon but may retain short-range tetrahedral coordination. Their electronic structure is influenced by nanoscale confinement, bond-angle disorder, hydrogen incorporation, and localized defect states. Their photoluminescence may therefore involve disorder-related or localized states rather than predominantly quantum-confined crystalline-core transitions. Atmospheric-pressure plasma experiments have shown that changes in silane concentration and particle heating can shift the product between predominantly amorphous and predominantly crystalline structures. Alessi et al. demonstrated close relationships among particle size, crystallinity, hydrogen incorporation, particle temperature, and electronic structure [50]. The results indicate that crystallization is not governed by pressure or precursor concentration alone, but by the thermal and chemical history experienced by particles during their residence in the plasma. Amorphous and partially crystalline SiQDs can be useful for low-temperature processing, direct film deposition, and applications in which tolerance to variations in operating conditions is more important than obtaining a highly ordered crystalline core. Their structural heterogeneity, however, complicates comparisons of optical performance. Reliable interpretation requires independent characterization of crystalline fraction, hydrogen content, oxidation state, defect density, and surface composition [22,41,50,51,52].

#### 2.1.3. Core–Shell SiQDs

Core–shell SiQDs consist of a crystalline or silicon-rich core surrounded by a chemically distinct passivating layer. The most widely investigated examples are Si/SiO_2_ and Si/SiN_x_ nanocrystals. A well-developed shell can reduce the density of nonradiative surface defects, slow uncontrolled environmental oxidation, and modify carrier localization at the core–shell interface. High-pressure water-vapor annealing has been used to convert hydrogen-terminated SiQDs into Si/SiO_2_ core–shell structures while preserving tunable photoluminescence. Loh et al. showed that thicker and more structurally relaxed silica shells produced environmentally stable PLQYs above 40% under the storage conditions investigated [24]. Shell thickness and optical response nevertheless depend on the original particle size, oxidation conditions, and structural quality of the Si/SiO_2_ interface. Si/SiN_x_ core–shell nanocrystals have been prepared through sequential nonthermal-plasma processing. In this approach, Si cores formed in an initial plasma zone are subsequently exposed to nitrogen-containing reactive species, producing a nitride-rich surface layer [53]. The shell can enhance photoluminescence and reduce the sensitivity of the Si surface to oxidation. At the same time, incomplete coverage, interfacial strain, partial oxidation, and trap formation may introduce additional nonradiative pathways. The final optical properties therefore depend on both shell composition and interface quality.

#### 2.1.4. Matrix-Embedded SiQDs

Matrix-embedded SiQDs are nanoscale silicon domains dispersed within a continuous dielectric or semiconductor host. Common matrices include SiO_2_, SiN_x_, SiC, and silicon-rich amorphous films. These structures are commonly fabricated by PECVD, sputtering, or deposition of silicon-rich layers followed by thermal annealing [51,52]. This architecture differs fundamentally from that of discrete core–shell nanoparticles. In an embedded system, the Si nanocrystals form part of a continuous solid film and cannot normally be isolated without disrupting the host. Their optical response depends on the size and crystallinity of the Si domains, matrix band alignment, strain, interfacial bonding, and host-related defects. Remote PECVD has been used to form Si-rich nanodomains within SiC films, with the CH_4_/H_2_ ratio affecting composition, bonding structure, nanocrystal formation, and luminescence [51]. Matrix confinement generally provides higher thermal and mechanical stability and greater resistance to oxidation than many colloidal SiQD systems. These properties make embedded SiQDs attractive for integrated optoelectronics, solid-state light emitters, optical switching, and luminescent solar concentrators [51,52]. The origin of emissions in embedded systems must nevertheless be interpreted carefully. Quantum-confined Si-core transitions can overlap with defect- and interface-related emission from the surrounding matrix, and the relative contribution of each pathway depends on preparation and annealing conditions. Such a material has been named polymorphous silicon (pm-Si:H) and extensively studied.

#### 2.1.5. Surface-Functionalized SiQDs

The high surface-to-volume ratio of colloidal SiQDs makes their optical and colloidal properties highly sensitive to surface termination. Hydrogen-terminated particles can be modified through hydrosilylation, whereas oxide-containing surfaces can be functionalized through silanization or related coupling reactions. Alkyl, carboxyl, amine, and polyethylene glycol-containing ligands are commonly used to improve colloidal stability, water dispersibility, molecular recognition, and compatibility with aqueous or biological environments [6,54,55,56].

Surface functionalization affects more than solubility. Ligand identity, binding configuration, surface coverage, residual Si–O bonds, structural strain, and incompletely passivated dangling bonds can alter radiative and nonradiative recombination. It is therefore too simplistic to assume that short-chain ligands always preserve PLQY or that longer and more polar ligands inevitably quench emissions. Ligand effects must be considered together with core size, surface coverage, oxidation state, structural relaxation, and defect density [55,56]. Ligand coverage can also affect charge transport and electroluminescence even when changes in ensemble PLQY are comparatively modest [56]. This distinction is important when comparing SiQDs intended for solution-phase sensing with those incorporated into light-emitting devices. Microplasma-assisted electrochemical synthesis provides an example in which nanoparticle formation and surface modification occur within a closely coupled process. The resulting SiQDs contained abundant surface functionalities, remained stable in colloidal dispersion, and enabled label-free dopamine detection over a broad concentration range [54].

For biomedical applications, the term biocompatible should be used with appropriate qualification. Hydrophilic or PEG-containing ligands can improve dispersion and reduce nonspecific interactions, but biological response also depends on particle size, dose, surface charge, ligand chemistry, residual synthesis products, aggregation state, exposure duration, and the biological model used for evaluation [6].

### 2.2. Carbon Quantum Dots

Carbon dots are more structurally heterogeneous than conventional crystalline semiconductor QDs. Materials with similar particle sizes and fluorescence colors may contain different combinations of graphitic domains, amorphous carbon, polymer networks, molecular fluorophores, defects, and surface states. Particle size alone is therefore insufficient for classification. Structural assignments should be supported by complementary microscopic, spectroscopic, chromatographic, and chemical evidence [3,43,44].

#### 2.2.1. Graphitic Carbon Dots

Graphitic CQDs contain nanoscale sp^2^-rich carbon domains embedded within or surrounded by less ordered carbon and oxygen- or nitrogen-containing surface groups. Their diameters commonly lie within the 2–10 nm range, although the extent and arrangement of graphitic ordering are generally more informative than particle size alone. HRTEM may reveal lattice fringes consistent with local graphitic ordering. Spacings near 0.21 and 0.34 nm are frequently associated with in-plane graphitic organization and interlayer stacking, respectively, although their exact assignment depends on the structure of the investigated sample. Raman spectroscopy, XPS, XRD, electron diffraction, and solid-state spectroscopic methods are often required to distinguish graphitic domains from predominantly amorphous carbon with only short-range ordering. Photoluminescence may involve transitions within π-conjugated carbon domains, but core states commonly coexist with edge, surface, and defect-related states. Excitation-dependent emission should therefore not automatically be attributed to a broad particle-size distribution. It may instead reflect several emissive centers with different local chemical environments and relaxation dynamics [3,43,44]. Graphitic CQDs are more accurately represented as compact nanoparticles containing interconnected or stacked sp^2^-rich domains than as hollow fullerene-like cages.

#### 2.2.2. Carbonized Polymer Dots

Carbonized polymer dots are formed through dehydration, condensation, cross-linking, and partial carbonization of molecular or polymeric precursors. Their structures contain cross-linked polymer-like regions together with carbonized domains whose degree of conjugation and graphitic ordering depends on precursor chemistry, reaction temperature, treatment time, and purification. Emission from CPDs may arise from molecular fluorophores, sub-fluorophores immobilized within cross-linked networks, partially conjugated carbon domains, surface states, or combinations of these centers. Their relative contributions can change as precursor conversion and carbonization progress. CPDs should therefore not be regarded as a structurally uniform class governed by a single emission mechanism. The confined-domain crosslink-enhanced-emission model provides an important explanation for selected CPD systems. Restricted intraparticle motion and interactions among luminophores can suppress nonradiative relaxation and modify fluorescence and phosphorescence. Tao et al. demonstrated this behavior in structurally confined carbonized polymer dots, providing direct support for crosslink-enhanced emission [57]. High PLQYs have been reported for selected CPDs, but they are not a defining property of the entire class. The measured efficiency depends on molecular-fluorophore formation, precursor ratio, cross-link density, degree of carbonization, aggregation, and purification.

#### 2.2.3. Graphene Quantum Dots

Graphene quantum dots are finite single- or few-layer graphene fragments with lateral dimensions commonly below approximately 10 nm. Unlike quasi-spherical graphitic CQDs, GQDs possess a predominantly two-dimensional architecture. Their electronic and optical properties are influenced by lateral confinement, layer number, edge topology, oxidation state, and functional groups located at edge or basal-plane sites [3,58,59]. GQDs can be prepared through top-down cutting or exfoliation of graphite, graphene oxide, carbon fibers, and related carbon materials, or through bottom-up assembly from aromatic molecular precursors. Direct structural observations of single-layered GQDs support their distinction from quasi-spherical carbon dots, while spectroscopic studies have demonstrated that edge structure and oxygen-containing functional groups can strongly affect their photoluminescence [58,59]. Graphitic CQDs and GQDs should therefore be treated as separate categories. The abbreviation GQD should be reserved for graphene quantum dots and should not be applied to quasi-spherical graphitic carbon dots.

#### 2.2.4. Heteroatom-Modified CQDs

Heteroatom incorporation is best regarded as a compositional modification rather than independent core architecture. Nitrogen, sulfur, phosphorus, boron, and combinations of these elements can be introduced into graphitic CQDs, CPDs, GQDs, or biomass-derived dots. These elements may occupy substitutional positions within sp^2^-rich domains or occur as edge and surface functionalities. Nitrogen-containing CQDs may contain pyridinic, pyrrolic, graphitic, amino, amide, or related nitrogen configurations, each of which affects the electronic structure differently. Nitrogen incorporation does not necessarily cause a red shift or increase PLQY. Castor-seed-derived N-CQDs, for example, exhibited predominantly blue emissions, demonstrating that nitrogen content alone does not determine emission color [45]. S, N-self-doped CQDs prepared from plant-derived precursors have been used for fluorescence detection of nitazoxanide and hemoglobin [46]. Phosphorus-doped CQDs have been applied as fluorometric probes for Fe^3+^ [47], while B, N-co-doped CQDs have exhibited red emission and lysosome-targeted imaging behavior [48]. These examples show that the final optical response depends on the interaction of dopant identity and bonding configuration with conjugation length, carbonization degree, defect density, core structure, and surface chemistry. Heteroatom incorporation can therefore modify electronic and surface states, tune emission, and increase PLQY in selected systems, but these effects are not universal.

#### 2.2.5. Biomass-Derived CQDs

Biomass-derived CQDs are classified by precursor origin rather than by unique internal architecture. Agricultural residues, fruit peels, leaves, seeds, plant extracts, shells, and food-processing byproducts can be converted into fluorescent carbon dots through hydrothermal treatment, microwave-assisted processing, or pyrolysis [27,28,60,61,62,63]. Natural precursors contain varying proportions of carbohydrates, proteins, amino acids, polyphenols, lipids, minerals, and other organic constituents. These compounds may act simultaneously as carbon sources, surface-passivating species, molecular-fluorophore precursors, and heteroatom donors. Biomass-derived CQDs are therefore often rich in oxygen-containing surface groups and may contain nitrogen or sulfur when these elements are present in the original feedstock. Biomass origin, however, does not automatically imply heteroatom self-doping. Incorporation of nitrogen, sulfur, phosphorus, or other elements should be demonstrated through elemental analysis and high-resolution XPS rather than inferred from precursor origin alone. Representative examples include CQDs prepared from banana peel, water hyacinth, *Aloe barbadensis*, castor seeds, and almond resin [28,45,60,61,62,63]. Such methods support waste valorization and reduce dependence on purified molecular precursors. Their principal limitation is feedstock variability. Moisture, mineral content, cultivation conditions, storage history, and biochemical composition can affect product yield, surface chemistry, particle-size distribution, and photoluminescence. Reproducible biomass-based synthesis therefore requires standardized precursor pretreatment, controlled reaction conditions, rigorous purification, compositional analysis, and batch-to-batch optical characterization.

### 2.3. Silicon Carbide Quantum Dots

Silicon carbide quantum dots form a hybrid class in which silicon and carbon are covalently bonded within cubic or hexagonal SiC lattices. They are more appropriately positioned between the SiQD and CQD families than classified as a subtype of carbon dots. Their wide bandgap, chemical resistance, thermal stability, and size-dependent electronic properties make them promising for harsh-environment sensing, high-temperature optoelectronics, nanocomposites, and selected biomedical applications [64,65]. Low-pressure nonthermal-plasma synthesis from tetramethylsilane has produced β-SiC nanocrystals with controlled dimensions of approximately 2–4 nm. These particles exhibited size-dependent changes consistent with quantum confinement, but no measurable photoluminescence was detected in that system. Oxide-containing surface groups promoted colloidal dispersion but may also have introduced nonradiative trap states [64]. Photoluminescence should therefore not be regarded as an intrinsic property of all plasma-synthesized SiC QDs. Blue-shifted emissions have been reported for selected SiC nanoparticles produced under different plasma and surface-chemistry conditions [65], illustrating the importance of synthesis route, crystal polytype, stoichiometry, particle size, carbon-rich domains, surface oxidation, and defect density. A general description applicable to the full SiC QD class is therefore that these materials exhibit size- and surface-dependent optical behavior.

### 2.4. Cross-Cutting Structure–Property Relationships

SiQDs and CQDs share several practically important characteristics, including nanoscale dimensions, tunable photoluminescence, high surface-to-volume ratios, and strong sensitivity to surface and interface chemistry. Nevertheless, their photophysical behavior differs substantially. In crystalline SiQDs, luminescence is primarily associated with quantum-confined core states modified by surface and interface effects, whereas CQDs often contain multiple coexisting emissive centers, including π-conjugated domains, surface and defect states, molecular fluorophores, and cross-linked structures [3,43,44,57,58,59]. Their optical response therefore cannot be described by particle size or elemental composition alone, but reflects the combined influence of core structure, crystallinity, surface chemistry, heteroatom configuration, defect states, and the local chemical environment [3,43,44,50,51,57]. Core–shell formation, ligand functionalization, matrix embedding, and heteroatom incorporation can further tune emission and stability, although these modifications may also introduce interfacial strain or nonradiative traps [6,24,45,46,47,48,49,50,51,52,53,54,55,56]. Reliable structure–property relationships consequently require complementary structural, chemical, and time-resolved optical characterization.

From a synthesis perspective, plasma-assisted and green routes provide distinctly different means of controlling these structural and optical features. Plasma provides additional control knobs over precursor dissociation, nucleation, particle growth, crystallinity, and surface passivation through multiple tunable discharge parameters, which can facilitate the synthesis of crystalline SiQDs and structurally well-defined nanomaterials under appropriately optimized conditions. In contrast, green synthesis relies predominantly on solution-phase chemistry, renewable or low-toxicity precursors, and thermal or hydrothermal conversion, offering simpler processing, lower environmental impact, and facile surface functionalization, particularly for CQDs. However, the broader chemical complexity of green routes can lead to greater heterogeneity in particle structure and emissive-state distributions. Thus, plasma-assisted synthesis generally favors structural and process control, whereas green synthesis emphasizes chemical versatility, sustainability, and scalability. Their relative suitability therefore depends on whether the target application prioritizes crystallinity and reproducibility or functional diversity and environmentally benign processing.

The central distinction is therefore not compositional but photophysical. For crystalline SiQDs, particle size remains a physically meaningful descriptor because confinement occurs within a defined semiconductor core. For CQDs, the TEM diameter may have little correspondence with the emissive domain because core states, surface states, and molecular fluorophores can coexist. Consequently, synthesis routes should be compared by their ability to control the relevant emissive structure, rather than by particle size alone.

## 3. Plasma-Assisted Synthesis of Silicon and Carbon Quantum Dots

Plasma-based synthesis occupies an intermediate position between conventional gas-phase processing and solution-based nanochemistry. In nonthermal plasma, energetic electrons efficiently excite, ionize, and dissociate molecular precursors, while the ions and neutral gas remain at considerably lower temperatures. This non-equilibrium energy distribution allows precursor activation and nanoparticle nucleation to proceed without the high bulk temperatures required in conventional thermal processes. It can also promote selective heating of growing nanoparticles and, in suitable reactor configurations, continuous particle production. In addition, the negative charging of nanoparticles can reduce coagulation and influence their confinement and residence time within the plasma.

These characteristics are particularly relevant to SiQDs, where crystallinity and surface termination strongly affect the electronic structure and photoluminescence, and to CQDs and GQDs, whose optical properties depend on a more complex combination of carbon-core structure, heteroatom incorporation, molecular fluorophores, and surface-related states [9]. However, plasma-assisted synthesis encompasses several distinct processing environments. Low-pressure radiofrequency (RF) reactors are commonly used for gas-phase formation of free-standing nanoparticles, whereas atmospheric-pressure plasma jets and confined microplasmas enable operation without vacuum systems and generally involve shorter particle residence times. Plasma–liquid systems introduce additional interfacial and solution-phase chemistry, while plasma-enhanced chemical vapor deposition (PECVD) is primarily used to form nanostructures within or on a solid matrix rather than as isolated colloidal particles.

These approaches should therefore not be considered equivalent. Their discharge characteristics, operating conditions, particle-formation pathways, product forms, and post-processing requirements differ substantially and should be evaluated using criteria appropriate to each synthesis route. For free-standing SiQDs, low-pressure continuous-flow RF/CCP reactors are treated here as the reference plasma architecture because they have the most mature evidence base for controlled nucleation, continuous particle production, size selection, collection, and downstream passivation. Atmospheric-pressure jets and confined microplasmas are discussed as emerging or specialized platforms and should not be interpreted as equivalent in throughput or technological maturity unless quantitative mass-yield and long-duration operating data are available. In the following sections, the different plasma-based approaches are therefore compared according to their synthesis environment, particle-growth mechanism, and resulting material properties rather than by reactor configuration alone [67].

### 3.1. Non-Equilibrium Chemistry, Nucleation, and Particle Growth

The key characteristic of a nonthermal plasma is its strongly non-equilibrium electron energy distribution function. Electron-impact dissociation, ionization, and attachment generate reactive radicals and ions while the bulk gas temperature remains well below that required for conventional thermal decomposition. During the synthesis of silicon quantum dots, silane (SiH_4_) or higher silicon hydrides, such as cyclohexasilane (Si_6_H_12_), are converted into SiH_x_ radicals, ions, and small silicon clusters. Once supersaturation is established, homogeneous nucleation occurs, followed by radical addition, surface reactions, coagulation, and, under favorable particle-energy conditions, crystallization. Exothermic surface recombination reactions can raise the temperature of an individual nanoparticle well above that of the surrounding gas. This localized heating provides a plausible explanation for the formation of crystalline silicon nanocrystals in reactors that remain at a relatively low bulk temperature [9]. Carbon-containing plasmas follow a broadly similar growth sequence, although their reaction chemistry is considerably more complex. Hydrocarbon precursors, most commonly methane (CH_4_) or acetylene (C_2_H_2_), undergo electron-impact dissociation and ionization to form CH_x_ radicals, C_2_ species, ions, and larger unsaturated molecular fragments. These reactive species subsequently polymerize, undergo aromatization, and form carbon-rich clusters. At a plasma–liquid interface, additional processes involving solvated electrons, radicals, ultraviolet radiation, interfacial electric fields, and localized heating occur simultaneously. Consequently, the resulting material may contain graphitic domains, amorphous carbon, molecular fluorophores, and oxygen- or nitrogen-containing surface groups. The classification of a nanoparticle as a carbon dot should therefore not rely on particle size alone. Evidence for a nanoscale carbon core should be supported by structural characterization and spectroscopic analysis capable of distinguishing core-state, surface-state, and molecular emission [68]. Particle charging strongly influences the growth of both silicon and carbon nanoparticles. Because electrons generally reach a growing particle more rapidly than positive ions, the particle acquires a negative floating potential. Electrostatic repulsion between similarly charged particles can suppress coagulation, while electric fields in the plasma sheath and ambipolar region can confine particles and extend their effective residence time. At higher particle number densities, however, charge fluctuations, ion-mediated attractive interactions, and partial neutralization can promote coagulation. Surface chemistry becomes increasingly important during the later stages of growth. Hydrogen termination can suppress non-radiative recombination through silicon dangling bonds, whereas controlled oxidation or nitridation can produce protective surface shells. Plasma-generated organic species may also provide direct in situ functionalization of freshly formed particles [9].

The kinetic picture of nanoparticle formation in low-pressure silane plasmas was established in the early studies of Bouchoule and Boufendi and refined in subsequent work [69,70]. In Ar/SiH_4_ discharges, particle formation proceeds from molecular clustering to the appearance of a relatively narrow population of nanometre-scale particles, followed by coagulation and surface-growth stages. Because the particles become negatively charged and can be electrostatically confined within the discharge, their effective growth time may differ considerably from the nominal gas residence time. Later studies demonstrated that the earliest stages of nucleation can be detected electrically before the particles become accessible to conventional light-scattering diagnostics. In particular, Boufendi et al. identified particles below approximately 5 nm through changes in the harmonic content of the RF discharge current [71]. Subsequent work showed that the first 2–4 nm crystallites formed in reactive plasmas may be treated as useful quantum-confined building blocks, provided that their nucleation, growth, and extraction are carefully controlled [72]. Changes in discharge power and pressure were found to shift the nucleation time and subsequent growth trajectory, while combined electrical and optical measurements provided complementary information on particle formation. Since no single diagnostic signal is uniquely determined by nanoparticle growth, reliable in situ monitoring is better achieved by combining several plasma observables with an independent particle measurement [73]. These studies established self-bias voltage, RF harmonic content, and electron-density variations as practical indicators of nanoparticle nucleation and growth in reactive plasmas.

**Figure 2:**
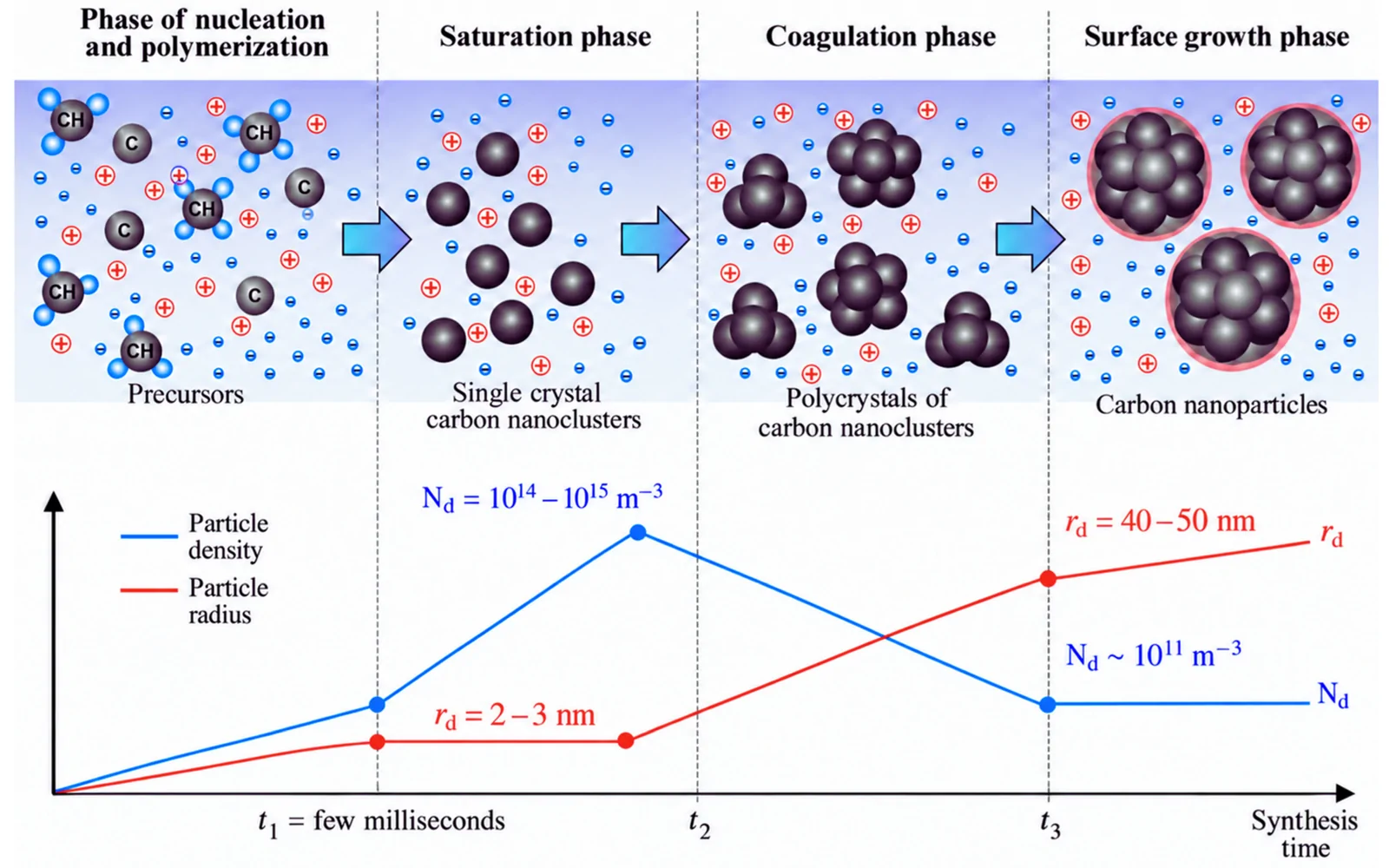
The mechanism of nanoparticle growth in RF discharge [74].

The RF plasma experiments reported by Orazbayev et al. provide a representative example of how electrical diagnostics can be correlated with carbon-particle growth in an Ar/CH_4_ capacitively coupled discharge [74]. The experiments were conducted in a 13.56 MHz reactor at pressures of 0.1–0.8 mbar and applied powers of 5–35 W. As illustrated in basic mechanism Figure 2, carbon-particle formation proceeded through four main stages: nucleation and polymerization, saturation, coagulation, and surface growth. At the beginning of the discharge (t = 0), reactive carbon-containing fragments underwent polymerization and formed the first nanoscale clusters. By the saturation stage, denoted as (t_1_), the clusters had grown to approximately 2–3 nm and began to agglomerate. Coagulation became dominant at (t_2_), producing larger particles with an average diameter of approximately 50 nm. During the subsequent surface-growth stage, reactive carbon-containing species continued to deposit on the existing particles. The particle number density remained approximately constant during this period, whereas the mean particle diameter continued to increase.

The evolution of the particle population was accompanied by measurable changes in the self-bias voltage and electron density. As the total particle surface area increased, electron losses to the particles became more pronounced, altering the electron balance and the electrical characteristics of the RF sheath. Although the final particles were larger than typical isolated fluorescent carbon quantum dots, the observed sequence of nucleation, polymerization, saturation, coagulation, and surface growth is directly relevant to controlling the transition from quantum-sized carbon clusters to larger carbon nanoparticles. More broadly, these results demonstrate that plasma electrical signals can be used as practical tools for process monitoring rather than being treated solely as descriptive discharge parameters.

### 3.2. Low-Pressure RF-CCP Synthesis of Quantum Dots and Nanoclusters

Low-pressure radio-frequency capacitively coupled plasma (RF-CCP) reactors are among the most established platforms for the gas-phase synthesis of silicon quantum dots and related ultrasmall silicon clusters. In a typical flow-through configuration, diluted silane or a volatile liquid-silane precursor passes through a quartz tube surrounded by external electrodes or intersected by internal RF electrodes. Particle formation depends on the combined influence of precursor supersaturation, electron density, pressure, residence time, hydrogen concentration, and particle temperature. Higher RF power generally increases precursor dissociation and nucleation, whereas longer residence times and higher precursor concentrations tend to promote particle growth. The effect of hydrogen is less straightforward because it simultaneously alters gas-phase chemistry, surface etching, passivation, and crystallization. Its influence therefore depends strongly on reactor geometry and particle thermal history. The development of continuous-flow plasma synthesis of luminescent Si nanocrystals was strongly shaped by the work of Mangolini, Kortshagen, and co-workers, who established low-pressure nonthermal plasma as a scalable route to free-standing luminescent Si nanocrystals [9,11]. Early work demonstrated high-yield continuous production [11], while subsequent studies achieved ensemble photoluminescence quantum yields exceeding 60% after controlled organic surface passivation [75] and further integrated aerosol-phase plasma synthesis with rapid in-flight processing and surface engineering [76]. Complementary studies by Veinot and co-workers demonstrated that nominally similar SiQD families can exhibit markedly different photoluminescence because core structure and surface chemistry are inseparable [41] and further clarified the role of surface groups in SiNC photophysics [77]. More recently, Kůsová and co-workers have provided detailed analyses of radiative lifetimes [38] and demonstrated the importance of distinguishing ensemble PLQY from the internal quantum efficiency of the emissive subpopulation [25]. Together, these contributions provide an important experimental framework for interpreting the synthesis–structure–surface–photophysics relationships discussed below.

The early studies of Bouchoule and Boufendi established the basic kinetic picture of particle formation in low-pressure Ar/SiH_4_ plasmas, describing the transition from molecular clustering to nucleation, electrostatic confinement, coagulation, and surface growth [69,70]. Boufendi et al. later showed that particles smaller than 5 nm could be detected through changes in the harmonics of the RF current [71]. This approach is particularly valuable for continuous processing because it identifies the onset of nucleation without interrupting the discharge or removing material from the reactor. Recent work demonstrates both the optical potential and the remaining limitations of RF plasma synthesis. Matějka et al. produced SiQDs with diameters starting from 2.4 nm and ensemble photoluminescence quantum yields (PLQYs) of up to 20% [25]. Spectrally resolved lifetime measurements indicated a near-unity internal quantum yield for the emissive subpopulation. This result should not be confused with the ensemble PLQY, since aggregation, structural defects, and incomplete surface passivation can leave a substantial fraction of particles optically inactive. The authors also showed that controlled-atmosphere collection and plasma-induced reactions in liquids can broaden the range of surface ligands while limiting uncontrolled oxidation of freshly formed SiQDs. Pringle et al. reported PLQYs approaching 70% for cyclohexasilane-derived silicon nanocrystals after oxygen-shielded size separation [39]. The result confirms the high optical quality achievable by plasma synthesis but also highlights the importance of downstream fractionation and air-free handling. Müller et al. obtained emission across the visible and near-infrared regions by varying synthesis and surface-modification conditions in a low-pressure RF flow-through reactor [78]. In a complementary approach, Hunter et al. used a dual-plasma system to form Si/SiN_x_ core–shell nanocrystals. After oxidation, the particles reached a PLQY of approximately 35% and showed improved resistance to environmental degradation [53]. These studies indicate that size control, collection, passivation, and purification must be treated as parts of the same process rather than as separate optimization steps. Low-pressure silane plasmas can also generate ultrasmall hydrogenated silicon nanoclusters that differ structurally from conventional diamond-like SiQDs. Their properties are determined not only by quantum confinement, but also by atomic topology, hydrogen termination, and electron delocalization. Multilevel simulations by Vach and Brulin showed that the balance between atomic and molecular hydrogen can steer early cluster growth toward amorphous structures, crystalline structures, or building units associated with hexagonal silicon nanowires [79,80]. In this regime, hydrogen affects collision dynamics, dangling-bond removal, and the stability of reactive intermediates, rather than acting only as a passivating species. Vach subsequently proposed an ultrastable class of approximately 1 nm non-tetrahedral silicon nanocrystals containing overcoordinated Si atoms and strongly delocalized electrons [81]. Despite their small size, these clusters were predicted to absorb across the ultraviolet, visible, and infrared regions. This model shows that plasma-generated Si clusters cannot always be treated as nanoscale fragments of bulk diamond silicon. At near-nanometer dimensions, topology and delocalized bonding may influence stability and optical behavior as strongly as conventional quantum-confinement effects. Experimental evidence reported by Jardali et al. showed that hydrogenated SiNCs formed in a pulsed silane plasma can possess a permanent electric dipole moment [82]. The dipole enabled electric-field alignment and catalyst-free sequential assembly of exceptionally thin silicon nanowires. Deposition of the same clusters onto LaB_6_ cathodes increased the thermionic current density by approximately a factor of 30 and improved resistance to chemical and mechanical degradation. Follow-up work demonstrated that field-induced association could form silicon superclusters with substantially larger collective dipole moments [83]. Plasma synthesis can therefore control not only particle size and surface composition, but also directional properties relevant to assembly and device integration. The final cluster structure also depends on the deposition process. Atomistic simulations by Ning and Vach showed that energy and substrate temperature determine whether a hydrogenated silicon cluster is reflected, softly deposited, restructured, or driven into a crystalline silicon surface [84]. Le et al. further predicted that oblique incidence can promote epitaxial incorporation by redistributing the impact energy and creating a transiently disordered region near the substrate surface [85]. The structure generated in the plasma may therefore be preserved or substantially altered during deposition. Low-pressure RF plasmas consequently provide access to two related but functionally distinct groups of silicon nano-objects. Conventional luminescent SiQDs are usually assessed in terms of diameter, crystallinity, surface chemistry, emission wavelength, lifetime, and PLQY. Hydrogenated dipolar SiNCs intended for field-directed assembly, thermionic cathodes, nanoelectronic interfaces, or proposed GHz-THz oscillators require additional descriptors, including atomic topology, permanent dipole moment, orientation, impact energy, and collective coupling [82,83,84,85,86]. Because these materials rely on different structure–property relationships, direct comparison using a single set of performance metrics may be misleading.

### 3.3. Atmospheric-Pressure RF Jets and Confined Microplasmas

Atmospheric-pressure jets and confined microplasmas provide compact platforms for mechanistic studies and specialized nanoparticle synthesis, but for free-standing SiQDs their reported material throughput, collection efficiency, and sustained-operating performance are generally less mature than those of established low-pressure continuous-flow RF systems. It also introduces short residence times, strong spatial gradients, and a greater tendency for precursor deposition on reactor walls. The HelixJet reported by Dworschak et al. is an atmospheric-pressure RF plasma source rather than a microwave reactor and should be classified accordingly [49]. Using He/Ar/H_2_/SiH_4_ mixtures, the authors produced silicon particles ranging from approximately 3 nm to more than 100 nm. Higher RF power and hydrogen addition promoted crystallinity and stronger photoluminescence, while an in-flight annealing zone provided further downstream crystallization. The broad accessible size range is useful for process exploration, but it also highlights the difficulty of maintaining a narrow quantum-dot distribution under atmospheric-pressure conditions. Confined microplasmas provide a different route to atmospheric-pressure control. Alessi et al. synthesized amorphous and crystalline SiQDs in a submillimetre plasma gap and obtained mean particle sizes of 1.5–5.5 nm with optical bandgaps of 2.3–2.5 eV [50]. Optical-emission measurements combined with collisional modelling showed that particle-surface temperature, rather than bulk gas temperature alone, determined whether crystallization occurred. This result points to a general design principle: atmospheric-pressure reactors should be compared in terms of local energy deposition and particle thermal history, not simply by applied power. Remaining practical challenges include nozzle fouling, sensitivity to flow perturbations, and the limited spatial separation of nucleation, growth, and annealing zones within a compact source. Accordingly, many atmospheric-pressure SiQD studies are best regarded as proof-of-concept demonstrations unless the product mass recovered, collection yield, precursor conversion, and continuous operating time are reported. This limitation should not be generalized to plasma–liquid CQD systems, which involve different formation mechanisms and are evaluated separately in Section 3.4.

### 3.4. Plasma–Liquid and Solution-Plasma Routes for Colloidal Si/C Quantum Dots

Plasma–liquid systems are especially attractive when the desired product must be water-dispersible or chemically functional immediately after synthesis. Depending on the reactor design, the discharge may contact the liquid directly, operate above the liquid surface, or form between submerged electrodes. Electron transfer, radical chemistry, interfacial electric fields, solvent decomposition, and local heating occur at the same time, allowing particle formation and surface functionalization to be combined in a single process. This chemical complexity is also the main limitation. Products obtained from organosilane or organic precursors may contain silicon-rich cores, silica-like domains, carbonaceous fluorophores, and covalently bound molecular species, making the origin of the observed luminescence difficult to assign to a single pathway. Chang et al. used a DC argon microplasma electrochemical reactor to synthesize surface-functionalized SiQDs from [3-(2-aminoethylamino) propyl] trimethoxy silane under ambient conditions [54]. The resulting colloids emitted blue light near 420 nm, remained stable in dispersion, and were applied to dopamine sensing. Ma et al. combined N-(β-aminoethyl)-γ-aminopropyltrimethoxysilane (DAMO) with citric acid in a plasma electrochemical process and increased the reported QY from 4.23% to 23.9%, with emission at 452.5 nm under 370 nm excitation [87]. Because amidation and condensation generated fluorescent molecular structures, this system is better regarded as a hybrid plasma-assisted organosilicon/carbonization route than as a purely gas-phase Si nanocrystal process. A different strategy is to synthesize the SiQD core upstream and functionalize it in the afterglow. Lee et al. introduced O_2_, water vapor, and acrylic acid into the downstream region of nonthermal plasma, thereby coupling controlled oxidation with ligand grafting [88]. The resulting water-soluble SiQDs emitted near 830 nm after aging, and their PLQY increased to approximately 30% after 8 days. The time dependence is scientifically important: aging may improve passivation, but it also means that PLQY values should be reported together with storage time, atmosphere, and measurement conditions.

Plasma-assisted synthesis of carbon-based quantum dots has increasingly moved toward gas–liquid and liquid-phase configurations, where short-lived reactive species generated by the discharge can directly participate in carbonization and surface functionalization [89]. Yang et al. reported size-selective formation of GQDs in organosulfate micelles using microplasma, with solvated electrons proposed to contribute to nucleation and growth [90]. Carolan et al. produced nitrogen-doped CQDs within minutes using an atmospheric-pressure microplasma and subsequently incorporated the particles into photovoltaic devices [91]. In-liquid plasma processing has also been applied to simple carbohydrate precursors; Treepet et al. showed that glucose, fructose, and sucrose yielded fluorescent carbon dots with differences in carbon-core ordering and metal-ion sensing behavior [92]. More recently, Pho et al. used a microplasma-jet/gas–liquid system with folic acid and demonstrated production rates approaching the gram-per-day scale [93]. These results indicate that plasma–liquid routes have genuine potential for increasing CQD productivity, although meaningful assessment of scale-up should consider not only fluorescence output, but also purification losses, recovered dry mass, energy demand, absolute PLQY, batch-to-batch reproducibility, and long-term reactor stability.

### 3.5. Inductively Coupled and Microwave Plasmas: Potential and Evidence Gaps

Inductively coupled plasma (ICP) and microwave sources can generate high electron densities without placing powered electrodes directly in the reaction zone. In principle, this geometry offers efficient precursor dissociation, lower electrode contamination, and a wider operating window for continuous flow. Microwave sources can also be implemented in compact atmospheric-pressure systems. These characteristics make both source classes attractive for high-throughput Si/C quantum-dot production [94].

Direct and quantitatively comparable studies of ICP- and microwave-synthesized SiQDs or CQDs are nevertheless less developed than the literature on RF-CCP, atmospheric microplasma, and plasma–liquid systems. Claims that ICP sources necessarily produce smaller dots, narrower distributions, or gram-per-hour yields cannot be generalized without matched precursor chemistry, residence time, collection efficiency, and PLQY measurements. The HelixJet is an RF-powered atmospheric-pressure source and is therefore considered here within the RF category rather than among microwave reactors [49]. More broadly, the present literature does not yet support a quantitative ranking of RF, ICP, and microwave sources because differences in precursor chemistry, residence time, collection efficiency, and post-synthesis processing often exceed differences attributable to excitation mode alone. At present, ICP and microwave reactors are best described as high-density plasma platforms with clear engineering potential but insufficient standardized benchmarking for a definitive performance ranking [9,10]. A meaningful reactor benchmark therefore requires more pressure and applied power. At minimum, studies should report precursor feed rate and conversion/utilization efficiency; recovered dry-particle mass, production rate, and collection efficiency; gas flow and residence-time distribution; specific electrical energy consumption per unit mass of recovered product; particle size distribution and crystalline fraction; surface/passivation state; PLQY measured on the same collected and purified fraction; and stability of these quantities during sustained operation. Without this information, apparent differences between RF-CCP, ICP, and microwave sources may reflect collection, post-processing, or precursor chemistry rather than the plasma excitation mode itself.

### 3.6. Plasma-Enhanced Chemical Vapor Deposition and Matrix-Embedded Quantum Dots

PECVD produces a fundamentally different product class: quantum dots embedded within a dielectric or semiconductor matrix. A silicon-rich SiO_x_, SiN_x_, or SiC film is deposited, after which thermal annealing or plasma-assisted phase separation forms nanocrystals within the solid [16,51,95]. The matrix suppresses oxidation and aggregation, provides mechanical stability, and is compatible with thin-film device fabrication. The trade-off is that the particles cannot be dispersed or functionalized independently, and their photoluminescence may include contributions from matrix defects and interface states. For plasma-borne hydrogenated clusters, the deposition event itself can be structure-selective. Molecular-dynamics studies by Ning and Vach and by Le, Jardali, and Vach showed that energy, incidence angle, and substrate temperature control whether a cluster undergoes soft landing, fragmentation, local restructuring, or epitaxial incorporation [84,85]. Particle properties measured in the gas phase therefore cannot automatically be assigned to the final film without considering the cluster-surface collision history. León-Guillén et al. used remote PECVD to grow SiC films and identified CH_4_/H_2_ conditions that reproducibly generated SiQDs after annealing at 600 °C [51]. The embedded dots produced blue emission with a maximum between 439 and 465 nm and strongly increased the integrated photoluminescence of the films. These results are relevant to LEDs and high-temperature photonics, but they should not be compared directly with the PLQY of colloidal, ligand-passivated SiQDs. More appropriate metrics include external emission efficiency, thermal stability, electrical transport, and compatibility with device processing. Plasma-grown SiC nanocrystals and Si/SiN_x_ core–shell particles further illustrate how carbon or nitrogen incorporation can provide robust inorganic passivation, although composition and interface defects must be carefully controlled [53,64].

**Figure 3:**
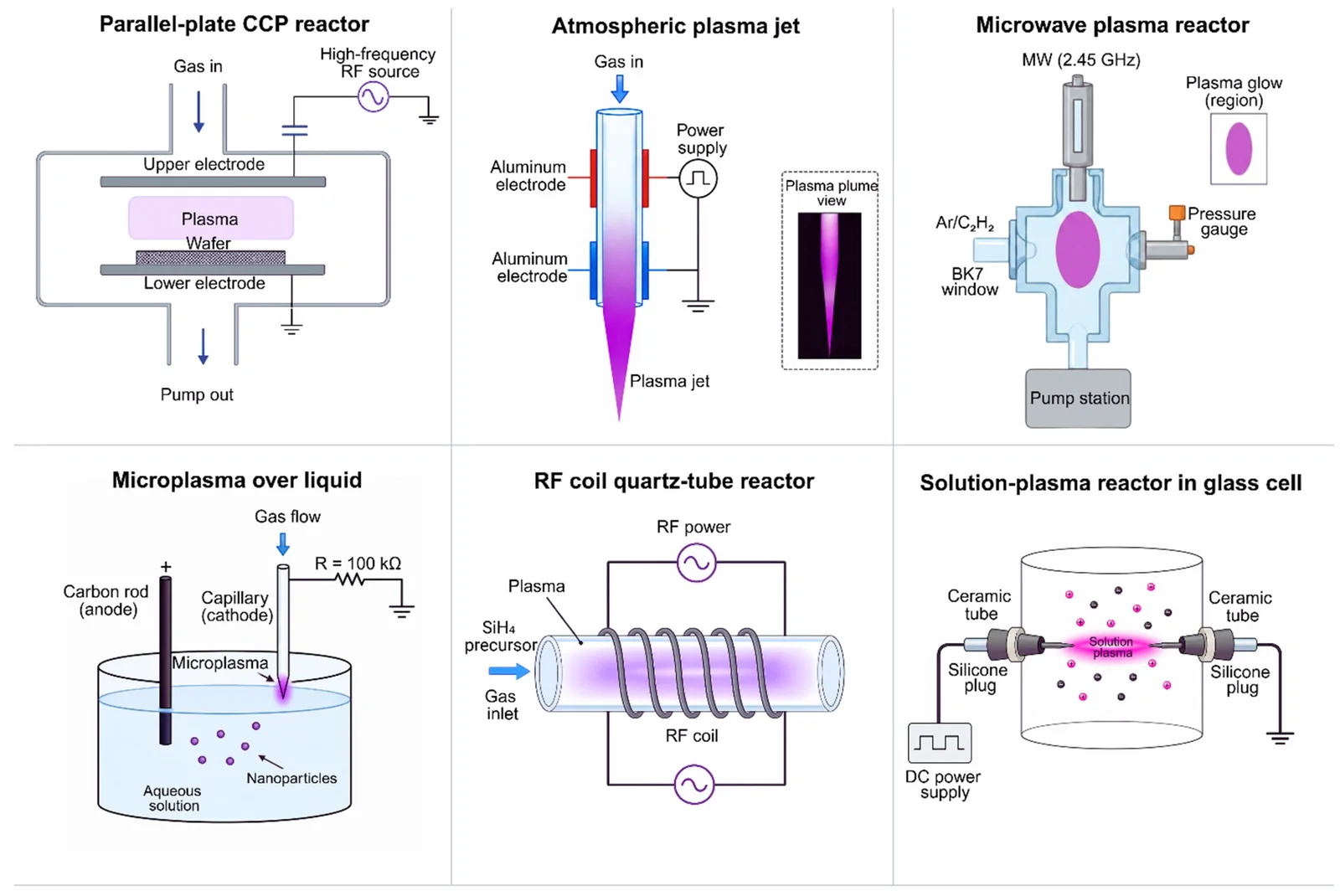
Representative plasma reactor configurations for the synthesis of silicon- and carbon-based quantum dots and related nanomaterials.

Overall, plasma synthesis (Figure 3) is most valuable when its non-equilibrium character is used deliberately rather than as a substitute heat source. Gas-phase charging can preserve small clusters, localized particle heating can crystallize SiQDs without heating the entire reactor, and plasma–liquid interfaces can couple nucleation with surface functionalization. Further progress will depend on combining these advantages with quantitative particle balances, operando diagnostics, reproducible post-processing, and clear distinctions between silicon nanocrystals, hybrid organosilicon dots, carbon dots, and larger plasma-grown carbon nanoparticles.

## 4. Green and Solution-Phase Synthesis of Carbon and Silicon Quantum Dots

Green and solution-phase methods complement plasma synthesis by providing direct access to colloidal quantum dots, broad precursor flexibility, and surface chemistries suited to sensing, bioimaging, catalysis, and solution-processed optoelectronics. Chemistry, however, differs substantially between carbon and silicon systems. Carbon quantum dots (CQDs) are commonly obtained by partial carbonization of molecular or biomass-derived precursors [96], whereas silicon quantum dots (SiQDs) generally require the conversion of silicon-rich precursors, chemical or electrochemical etching, laser ablation, and subsequent surface passivation [95,97]. These routes should therefore not be treated as a single mechanistic family.

The term “green” also requires careful use. Water or biomass alone does not make a synthesis sustainable. Heating demand, reagent toxicity, solvent replacement, dialysis-water consumption, product yield, and treatment of discarded fractions all contribute to the environmental burden. In this section, green synthesis refers to processes designed to reduce hazardous inputs and waste, solution-phase routes that may still involve high-temperature conversion, corrosive etchants, organic ligands, or extensive purification [8,34].

This distinction is particularly important when solution-phase synthesis is compared with gas-phase growth. In RF plasma experiments, the evolution of carbon particles has been followed in real time through changes in self-bias voltage and electron density [74]. Solution-phase syntheses rarely provide an equivalent in situ signature, and reaction progress is usually reconstructed from ex situ spectroscopy and microscopy. Strong fluorescence in a crude product is therefore not sufficient evidence for a chemically uniform population of quantum dots.

### 4.1. Reaction Design and Criteria for Sustainable Synthesis

**Figure 4:**
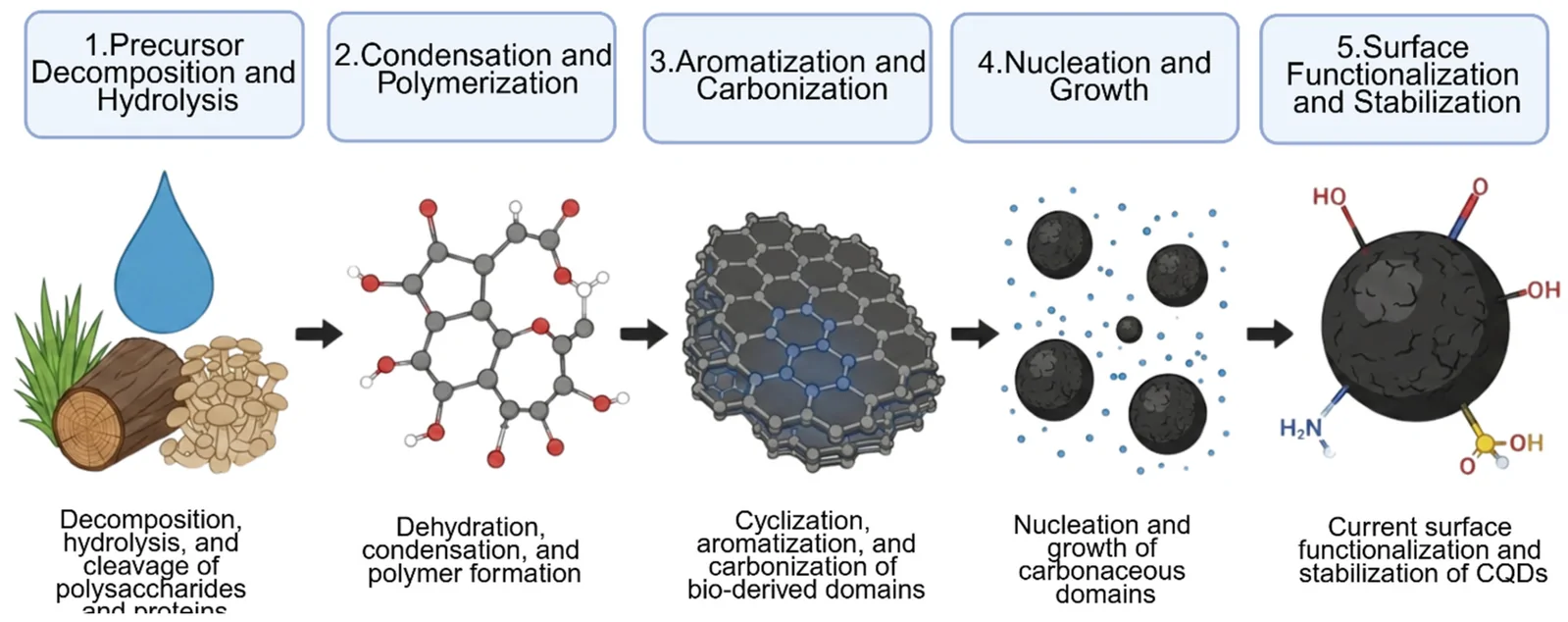
Conceptual formation pathway of biomass-derived carbon quantum dots during bottom-up hydrothermal or microwave-assisted conversion.

Bottom-up CQD synthesis begins with dehydration, condensation, and polymerization of oxygen- and nitrogen-containing molecules [98]. Further heating promotes cyclization and aromatization, often producing a mixture of carbonized nanoparticles, polymer-like domains, soluble oligomers, and molecular fluorophores. Although the detailed reaction network depends on precursor composition and processing conditions, bottom-up conversion of biomass-derived feedstocks can be represented by a sequence involving precursor decomposition and hydrolysis, dehydration and condensation, polymerization, aromatization and carbonization, nucleation of carbonaceous domains, and concurrent surface functionalization, as illustrated in Figure 4. Hydrolysis and decomposition of polysaccharides, proteins, and other biomass constituents generate smaller reactive species that undergo dehydration, condensation, and polymerization. Progressive cyclization, aromatization, and carbonization produce conjugated carbonaceous domains, followed by nucleation and growth of nanoscale particles. Oxygen-, nitrogen-, and sulfur-containing groups originating from the precursor or reaction medium contribute to surface termination and colloidal stabilization.

**Figure 5:**
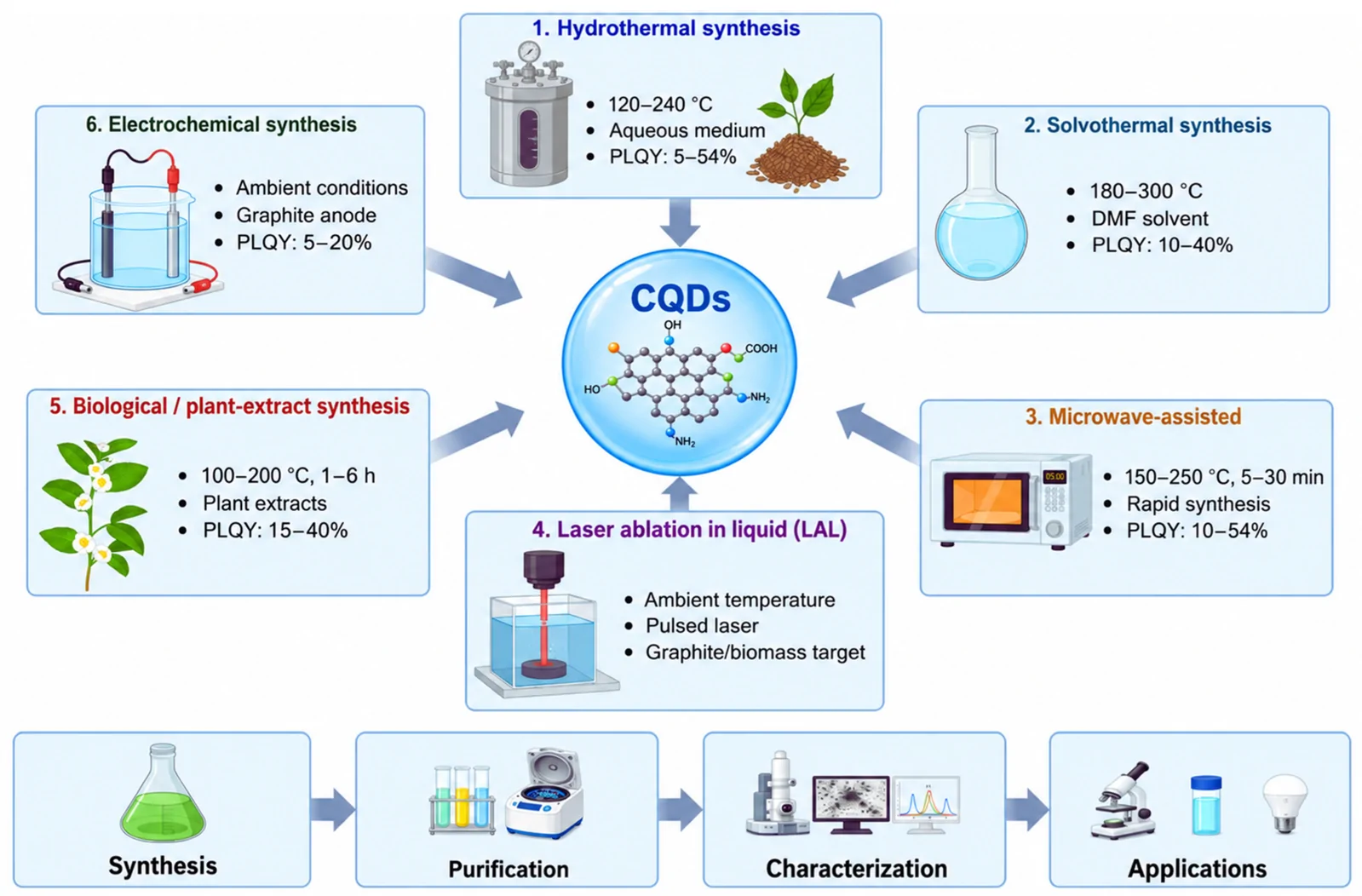
Major green and solution-based synthesis pathways for carbon quantum dots (CQDs).

Their relative abundance depends on precursor functionality, pH, solvent polarity, the temperature-time profile, and heteroatom availability [99]. Terms such as carbon dot, carbon nanodot, and carbonized polymer dot should therefore be assigned only after structural and fraction-resolved characterization, rather than based on blue fluorescence alone [100]. The principal green and solution-based routes used for CQD preparation, together with the common post-synthetic workflow, are summarized in Figure 5.

SiQD synthesis follows a different sequence. A silicon-rich solid or molecular precursor is first converted into nanoscale silicon domains, which are then released by etching or dispersed through laser or electrochemical processing [101]. The resulting H-terminated or oxidized surfaces must be stabilized by hydrosilylation, controlled oxidation, or ligand exchange. Surface chemistry is integral to synthesis because it governs colloidal stability, oxidation resistance, emission efficiency, and compatibility with biological or electronic environments [102].

### 4.2. Hydrothermal and Solvothermal Routes to Carbon Quantum Dots

Hydrothermal treatment is one of the most widely used bottom-up routes to CQDs because it requires simple equipment and tolerates a broad range of precursors [27]. An aqueous precursor is sealed in a polytetrafluoroethylene-lined autoclave and heated, typically at 120–240 °C for several hours. Autogenous pressure keeps the medium liquid above the normal boiling point of water and accelerates hydrolysis, dehydration, and condensation. Although the procedure is operationally simple, the actual thermal history depends on autoclave volume, filling fraction, heating rate, holding time, and cooling protocol.

Biomass-derived studies illustrate both the potential and the variability of this approach. Atchudan et al. converted banana-peel waste at 180 °C for 12 h into CQDs with an average size of approximately 5 nm and a reported photoluminescence quantum yield (PLQY) of 20%; the particles were also used for in vivo imaging in nematodes [28]. Under the same nominal temperature and reaction time, Zhao et al. prepared water-dispersible CQDs from water hyacinth. The hydroxyl- and carboxyl-rich material enabled Fe^3+^ detection with a reported limit of detection of 0.084 μM and fluorescence imaging of *Klebsiella* cells [60]. These examples show that biomass can supply both carbon and surface heteroatoms, but they do not imply that different feedstocks produce equivalent structures or optical properties.

Hydrothermal chemistry can also introduce analyte-responsive surface functionality. CQDs prepared from pomelo peel, for example, were used in a fluorescence turn-off/turn-on assay for Fe^3+^ and L-cysteine [103]. Such sensing behavior arises from surface coordination, charge transfer, and fluorescence quenching or recovery, and should not automatically be interpreted as evidence of intrinsic quantum-confinement emission.

Solvothermal synthesis uses high-boiling organic media such as ethanol, glycerol, dimethylformamide, or mixed solvents [104]. The wider temperature window and altered solvation environment can improve precursor conversion, tune surface polarity, and favor red-shifted emission. These benefits come with a higher solvent burden and more demanding purification. In particular, dimethylformamide-based routes should not be described as green without solvent recovery and waste analysis [105]. Hydrothermal processing is generally better suited to aqueous bioapplications, whereas solvothermal processing is useful when organic dispersibility, extended conjugation, or device integration is required.

### 4.3. Microwave-Assisted Carbonization

Microwave irradiation couples directly to polar molecules and ionic species, enabling rapid volumetric heating. In small batches, reactions that normally require several hours can often be completed within minutes. The principal advantage is therefore kinetic rather than mechanistic: dehydration, polymerization, and carbonization still occur, but the heating profile is much steeper and may include localized hot spots. Reproducible studies should report the reactor type, nominal or absorbed power, vessel geometry, temperature and pressure control, precursor volume, and cooling procedure.

Qandeel et al. demonstrated a four-minute, one-pot microwave route using onion and cabbage as renewable sulfur and nitrogen sources. The resulting self-doped CQDs were applied to fluorescence sensing of nitazoxanide and hemoglobin [46]. Malavika et al. used microwave-assisted reflux of *Aloe barbadensis* Miller extract to obtain biocompatible CQDs for photocatalysis, cell imaging, and antiproliferative studies [61]. Praseetha et al. reported sub-10 nm CQDs with a relative PLQY of 61% after centrifugation, filtration, 24 h dialysis, and lyophilization; the quantum yield was determined relative to quinine sulfate, with excitation/absorbance evaluation at approximately 350–360 nm [62]. This value should therefore be interpreted in the context of the purified fraction and the relative-QY protocol rather than as a directly comparable absolute PLQY benchmark. Together, these reports show that microwave processing can rapidly generate application-ready surface functionality, although the resulting optical performance remains strongly dependent on purification, sample preparation, and compositional characteristics.

Scale-up is more difficult than the short reaction time suggests. Microwave penetration depth, dielectric loss, and vessel loading control temperature uniformity, so a protocol optimized for a few millilitres cannot simply be transferred to a larger vessel. Continuous-flow microwave systems with controlled residence times offer a more credible route to scale [106]. Purified product yield should also be reported because intense fluorescence in a crude product may arise partly from soluble molecular fluorophores.

### 4.4. Biomass-Derived Precursors: Opportunity and Reproducibility

Plant residues, food waste, natural gums, and aquatic biomass are attractive because they are inexpensive and chemically diverse. Their polysaccharides, proteins, polyphenols, organic acids, and minerals can contribute to carbonization, passivation, and heteroatom incorporation in a single step. The same complexity, however, is a major source of batch-to-batch variation [107]. Species, season, geographic origin, storage, drying, extraction, and particle size can alter both the effective carbon-to-nitrogen ratio and the concentration of fluorescent molecular products.

Biomass feedstocks should therefore be characterized as chemical inputs rather than identified only by common name. At minimum, studies should report moisture and ash content, extraction conditions, solid-to-liquid ratio, and major elemental composition [108]. Control experiments using representative sugars, amino acids, or polyphenols can help determine which components dominate particle formation and emission. Without such controls, claims of self-doping remain largely qualitative.

Waste valorization is most convincing when the entire process is considered. Chen et al. converted the pollutant Reactive Red 2 into fluorescent carbon nanodots, illustrating how an undesirable organic stream can be transformed into a functional material [109]. The environmental case is strongest when the precursor is genuinely diverted from a waste-treatment pathway, additional toxic reagents are avoided, and the purified product yield is sufficient to justify the energy and water used. A low-yield synthesis that consumes large volumes of dialysis water may offer little practical advantage despite relying on renewable feedstock.

### 4.5. Electrochemical and Laser-Ablation Routes

Electrochemical synthesis provides a top-down route to CQDs and graphene quantum dots through anodic oxidation or exfoliation of graphite, carbon fibres, and related electrodes. Particle formation is controlled by electrode potential or current density, electrolyte composition, pH, and treatment time. The process operates near ambient bulk temperature and can support continuous collection, but electrode erosion, electrolyte contamination, and broad size distributions remain important limitations. Filtration, dialysis, or chromatographic fractionation is often required before optical properties can be assigned to a defined nanodot population [8,27].

Laser ablation in liquids eliminates molecular reducing agents and can produce relatively clean colloids from carbon or silicon targets. A pulsed laser creates a transient plasma plume and cavitation bubble, followed by rapid cooling, nucleation, fragmentation, oxidation, and surface termination in the surrounding liquid. The method is valuable when ligand-free or minimally contaminated particles are required. Its principal limitations are low mass productivity, high energy consumption, capital cost, and sensitivity to laser fluence, pulse duration, repetition rate, target scanning, liquid depth, and bubble shielding.

For silicon colloids, Intartaglia et al. reported one-step laser production and oligonucleotide conjugation of SiQDs with an average size of approximately 4 nm [110]. More recently, optimization of femtosecond pulsed-laser ablation in water identified an effective fluence window and showed that persistent cavitation bubbles can reduce productivity at excessive fluence [111]. Laser ablation can therefore provide clean and controllable products, but reagent-free processing should not be equated with low energy demand or straightforward scale-up.

### 4.6. Solution-Phase Synthesis and Surface Engineering of Silicon Quantum Dots

Solution-phase SiQD synthesis is generally more demanding than CQD carbonization because elemental silicon domains must be formed and protected against oxidation. Common routes convert hydrogen silsesquioxane, triethoxysilane-derived polymers, or related silicon-rich precursors into Si nanocrystals by thermal disproportionation or pyrolysis. Chemical etching then releases the nanocrystals and can narrow their size distribution, after which hydrosilylation attaches alkyl, carboxyl, or other ligands to the Si-H surface. The final emission depends jointly on core size, crystallinity, oxide content, ligand coverage, and residual defect states.

Terada et al. developed a cost-effective route in which methanol controlled the structure of a hydrogen silsesquioxane polymer. After pyrolysis, etching, and dodecyl passivation, 3–4 nm SiQDs emitted in the 700–800 nm range with PLQYs of approximately 10–25% [112]. Zhou et al. used an inexpensive triethoxysilane precursor and optimized annealing and etching to obtain SiQDs with a PLQY above 50% in a polymer nanocomposite and near-unity internal quantum efficiency for the emissive subpopulation [26]. These results demonstrate the optical quality achievable by precursor design, while also showing why high-temperature conversion and corrosive etching must be included in sustainability assessments.

Surface conversion determines whether SiQDs remain dispersible and emissive in the intended medium. Özbilgin et al. prepared carboxyl-terminated, water-soluble SiQDs through thermal hydrosilylation of 10-undecenoic acid on H-terminated nanocrystals and demonstrated long-term colloidal stability and photothermal functionality [113]. Xu et al. systematically varied hydrosilylation time and ligand coverage, showing that surface-ligand density affects both photoluminescence and electroluminescence in SiQD light-emitting devices [56]. Core diameter alone is therefore an inadequate descriptor; ligand identity, surface coverage, oxidation state, and purification are equally important process variables.

Hybrid plasma–electrochemical methods lie at the boundary between plasma and wet-chemical synthesis. Ma et al. used an aminosilane precursor and citric acid in an atmospheric plasma-assisted solution process, increasing the reported PLQY from 4.23% to 23.9% by adjusting precursor concentration and reaction time [87]. The method offers rapid production of water-dispersible blue-emitting material, but the proposed amidation and condensation reactions raise an important identification issue: silicon-containing fluorescent molecules or polymeric species must be distinguished from quantum-confined elemental Si cores using high-resolution structural and compositional analysis.

### 4.7. Purification, Fractionation, and Mechanistic Assignment

Purification is one of the most underestimated stages in bottom-up carbon-dot synthesis. Dialysis is widely used because it is simple, but membrane molecular-weight cut-off values do not correspond directly to nanoparticle diameter, and equilibrium may require repeated solvent replacement [114]. Centrifugation and filtration remove large aggregates but do not separate fluorescent small molecules from nanoscale particles. Size-exclusion chromatography, high-performance liquid chromatography, electrophoresis, and preparative ultrafiltration provide better fraction resolution, although they reduce yield and increase process complexity [100,114].

Fractionation is required for mechanistic interpretation, not merely for product appearance. Small molecular fluorophores and soluble oligomers can exhibit high PLQY and may dominate ensemble emission even when TEM confirms the presence of nanoparticles [115]. Recent reviews have therefore identified separation and purification as central unresolved problems in carbon-dot photophysics [100,114]. Han et al. further showed that several molecular fluorophores can evolve during the formation of carbonized polymer dots and contribute cooperatively to the measured emission [116]. A broad fluorescence spectrum alone cannot establish whether the dominant emissive state belongs to a carbon core, a surface state, or a molecular species.

## 5. Structural and Optical Properties

**Figure 6:**
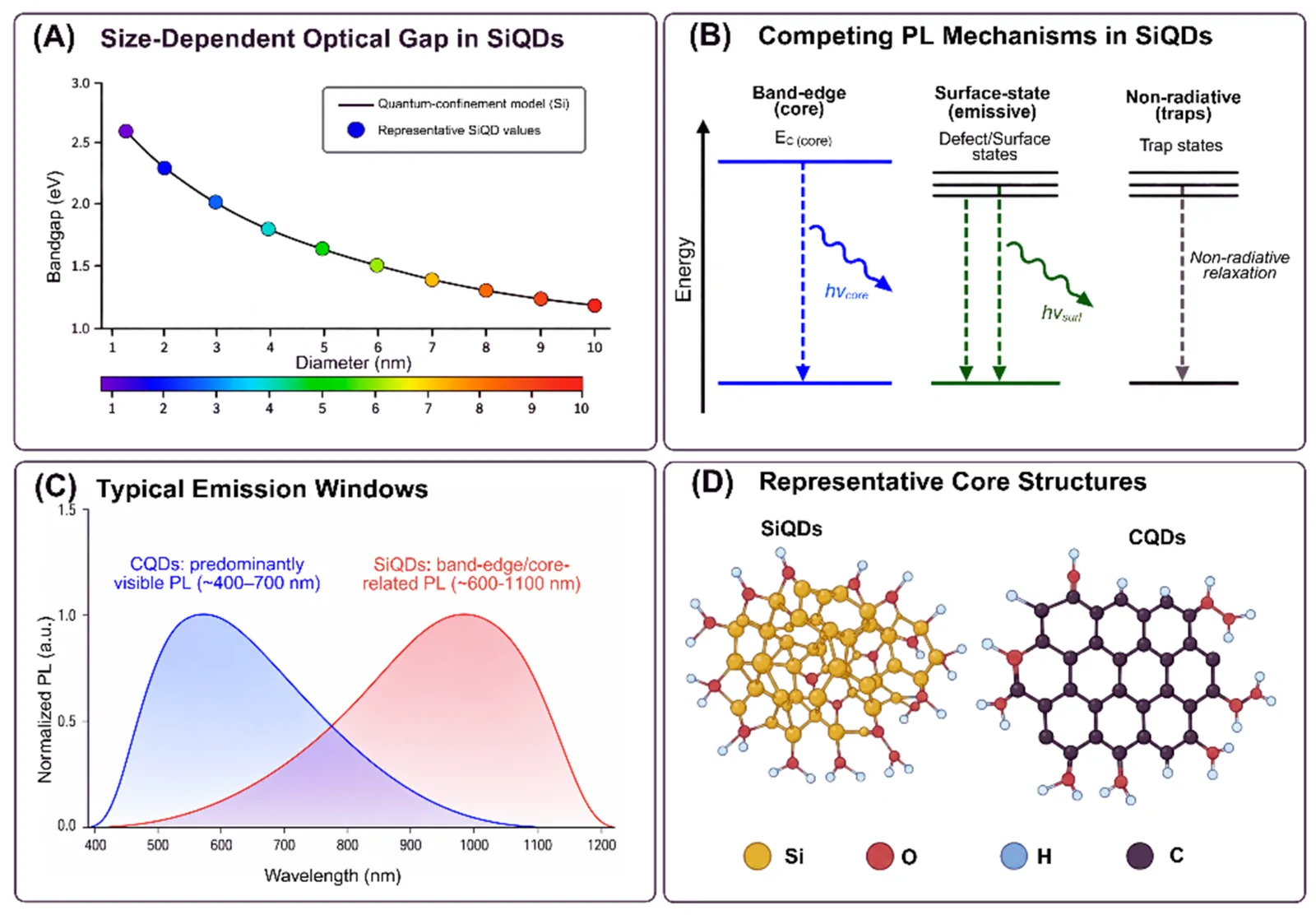
Comparative optical characteristics of silicon and carbon quantum dots: (**A**) size-dependent optical bandgap of SiQDs arising from quantum confinement; (**B**) competing photoluminescence pathways in SiQDs involving core-related, surface-state, and non-radiative trap-mediated processes; (**C**) representative emission windows of CQDs and SiQDs; and (**D**) representative core structures of SiQDs and CQDs.

Silicon quantum dots (SiQDs) and carbon quantum dots (CQDs) are often discussed using the same terms—quantum confinement, surface states, and photoluminescence quantum yield (PLQY)—but their microscopic photophysics are not equivalent. Crystalline SiQDs are quantum-confined semiconductor nanocrystals whose band-edge energies and recombination kinetics depend strongly on size, crystallinity, surface termination, oxidation, and the surrounding dielectric environment. CQDs comprise a more heterogeneous group that may include graphitic domains, amorphous carbon, carbonized polymer structures, surface-localized states, and molecular fluorophores. A meaningful comparison (Figure 6) therefore requires two complementary descriptions: a semiconductor-physics framework for SiQDs and a structure-resolved molecular/nanocarbon framework for CQDs [1,2,3,6,7].

### 5.1. Quantum Confinement and the Size–Bandgap Relationship

Quantum confinement is central to the optical behavior of semiconductor nanocrystals. When the nanocrystal dimensions become comparable to or smaller than the exciton Bohr radius, the spatial motion of electrons and holes is restricted, and the quasi-continuous electronic states of the bulk semiconductor evolve into discrete, size-dependent energy levels. The resulting increase in carrier confinement energy widens the effective optical bandgap. For silicon, the exciton Bohr radius is commonly estimated to be approximately 5 nm; pronounced size-dependent changes therefore emerge when the nanocrystal diameter is reduced to the few-nanometer regime [117,118].

Within the effective-mass approximation, the first-order dependence of the bandgap on nanocrystal size can be expressed as:

(1) $$E_{g}\left(R\right)\;\approx \;E_{g,bulk}\;+\;\frac{\hbar^{2}\pi^{2}}{2R^{2}}\left(\frac{1}{m_{e}^{*}}\;+\;\frac{1}{m_{h}^{*}}\right)\;-\;\frac{1.8e^{2}}{4\pi \varepsilon_{0}\varepsilon_{r}R}$$

where R is the nanocrystal radius, $m_{e}^{*}$ and $m_{h}^{*}$ are the effective masses of the electron and hole, respectively, and $\varepsilon_{r}$ is the effective relative permittivity of the nanocrystal and its surrounding dielectric environment. The $R^{2}$ term describes the increase in confinement energy, whereas the $R^{-1}$ term represents the Coulomb attraction between the confined electron and hole. Equation (1) captures the general tendency of the bandgap to increase as the nanocrystal becomes smaller. It should not, however, be used as a direct calibration curve for converting an emission wavelength into a particle diameter. The model assumes an approximately spherical particle, parabolic energy bands, ideal confinement, and a homogeneous dielectric environment. These assumptions become increasingly restrictive at very small dimensions, where atomistic structure, intervalley coupling, surface reconstruction, oxidation, ligand-induced strain, and dielectric mismatch may contribute as strongly as the nominal size effect [117,119].

In silicon quantum dots (SiQDs), a reduction in the crystalline-core diameter generally shifts band-edge emission from the near-infrared towards the visible region. Experimental spectra nevertheless show that particles of similar size can emit at different wavelengths. This variation arises because the electronic structure is also sensitive to crystallinity, surface termination, oxidation, interfacial strain, ligand chemistry, and the composition of the surrounding matrix. These factors affect not only the emission energy but also the balance between radiative and non-radiative recombination [117,120,121]. Particle size should therefore be evaluated together with structural and surface-sensitive characterization rather than inferred from photoluminescence spectra alone.

The interpretation is less straightforward for carbon quantum dots (CQDs). The diameter observed by transmission electron microscopy does not necessarily represent the dimensions of the actual emissive center. Depending on the precursor and synthesis conditions, the dominant emission may arise from a smaller sp^2^-conjugated domain, a surface-associated chromophore, a defect state, or a molecular fluorophore retained in the dispersion [3,43,57]. Consequently, an apparent correlation between CQD size and emission wavelength does not, by itself, demonstrate conventional quantum confinement. For CQDs, size-dependent spectral shifts should instead be considered alongside the degree of carbonization, the dimensions of the sp^2^ domains, surface composition, heteroatom content, molecular by-products, and sample purification. This combined interpretation is particularly important because CQD samples with comparable average particle sizes may display markedly different photoluminescence when their surface chemistry or molecular-state populations differ [3,43,57].

### 5.2. Photoluminescence Mechanisms in Silicon Quantum Dots

The photoluminescence of SiQDs reflects several competing radiative and non-radiative pathways (Table 2). In well-passivated crystalline particles, band-edge emission arises from recombination of quantum-confined carriers. Because silicon remains an indirect-gap semiconductor over much of the relevant size range, radiative recombination is commonly phonon-assisted and produces microsecond-scale lifetimes. These long decays distinguish SiQD core emission from the nanosecond fluorescence typical of many molecular emitters [39,56,120].

Surface and interface states introduce additional pathways. Dangling bonds, Si-O and Si-OH groups, incomplete ligand coverage, and ligand-induced interfacial states can create levels within the gap. Such states may provide radiative channels, but they can also act as efficient non-radiative traps. Their contribution depends on oxidation, ligand identity, surface coverage, and the local dielectric environment [56,121]. A faster quasi-direct or zero-phonon contribution has been reported for ultrasmall, carefully size-selected SiQDs. Strong confinement and surface modification can partially relax the indirect-gap selection rules, shortening the photoluminescence lifetime while maintaining high efficiency in well-passivated fractions [39,122]. This behaviour should not be described as a complete conversion of silicon into a conventional direct-gap semiconductor; phonon-assisted recombination, faster core-related channels, and surface losses evolve simultaneously as the particle size and surface chemistry change.

### 5.3. Photoluminescence Mechanisms in Carbon Quantum Dots

The emission mechanisms of CQDs are more heterogeneous than those of crystalline SiQDs, and no single model explains all reported samples. Three contributions recur throughout the literature: transitions within sp^2^-rich carbon domains, surface- or defect-localized states, and molecular fluorophores formed during bottom-up synthesis. Carbonized polymer dots may also exhibit cross-link-enhanced emission or charge-transfer states associated with heteroatom-rich polymeric networks [3,43,57].

Excitation-dependent emission is often attributed to a distribution of surface states, but it is not unique evidence for that mechanism. Similar behavior can arise from overlapping molecular fluorophores, chemically distinct particle fractions, reabsorption, or inner-filter effects. Conversely, excitation-independent fluorescence does not by itself prove a single carbon-core transition. Reliable assignment requires purification, excitation–emission mapping, time-resolved spectroscopy, and chemical identification of low-molecular-weight products in the same fraction used for optical measurements [3,43,57]. Precursor composition, reaction temperature, carbonization degree, surface passivation, and heteroatom doping all influence the balance between these emissive species. This structural diversity explains why nominally similar CQDs can display markedly different spectra, lifetimes, and PLQYs even when their mean particle sizes are comparable.

### 5.4. Photoluminescence Quantum Yield and Reporting

When comparing the optical performance of SiQDs and CQDs, particular care is required in the interpretation of photoluminescence efficiency. The photoluminescence quantum yield (PLQY), defined as the ratio of emitted to absorbed photons, is an ensemble property and therefore reflects the contribution of all absorbing species present in the measured fraction, including highly emissive particles, weakly emissive or non-emissive species, molecular byproducts, and aggregates. This quantity should be distinguished from the internal quantum efficiency of the emissive population, which may approach unity even when the measured ensemble PLQY remains substantially lower [25].

For plasma-synthesized SiQDs, ensemble PLQYs of around 20% have been reported for as-produced or broadly distributed samples, whereas size-selected and more effectively passivated fractions can reach considerably higher efficiencies [25,39]. Controlled formation of Si/SiO_2_ core–shell structures by water-vapour annealing has yielded PLQYs above 40% [24]. Values approaching 80%, together with light-emitting-diode external quantum efficiencies above 10%, have also been reported in a recent study [40]; however, such values should be interpreted with appropriate consideration of the reporting format and experimental details.

CQDs exhibit an even wider range of reported PLQYs because their emission may originate from a mixture of carbonized domains, surface- or defect-related states, and molecular fluorophores. Biomass-derived CQDs often show moderate efficiencies, whereas heteroatom incorporation, improved surface passivation, and the use of more structurally defined molecular precursors can substantially enhance their emission.

Meaningful comparison of PLQY values therefore requires careful reporting of the measurement protocol and sample history. Ideally, absolute PLQY should be determined using an integrating sphere, with the excitation wavelength, optical density, solvent, concentration, sample geometry, spectral correction, reabsorption treatment, replicate measurements, and uncertainty clearly documented. When relative quantum-yield methods are used, reference fluorophore, excitation wavelength, absorbance range, solvent, and refractive-index correction should also be reported. In addition, the purification or fractionation procedure and the identity of the measured fraction should be specified, since molecular fluorophores, oligomers, aggregates, or size-selected subpopulations may strongly affect the measured value. For synthesis studies, the purified material yield and PLQY should also refer to the same isolated fraction, since otherwise high optical efficiency may not reflect the performance of the overall synthesis process.

### 5.5. Absorption Properties and Stokes Shifts

SiQDs generally display broad ultraviolet-to-visible absorption that increases toward shorter wavelengths, often without the sharp excitonic peaks observed in highly monodisperse direct-gap nanocrystals. Size dispersion, surface oxide, and overlapping higher-energy transitions complicate the extraction of a single optical gap [120]. Core-emitting SiQDs can exhibit large Stokes shifts because absorption populates higher electronic states, whereas emission occurs after relaxation to the lowest confined exciton. This spectral separation is valuable in luminescent solar concentrators because it reduces, although does not eliminate, reabsorption losses [26]. CQDs also absorb strongly in the ultraviolet. Bands in the 200–280 nm region are commonly assigned to π–π* transitions in aromatic or graphitic domains, whereas weaker features between approximately 300 and 400 nm are often associated with n–π* transitions involving carbonyl or other heteroatom-containing groups [3,43]. These assignments are useful descriptors, but they do not establish the identity of the emissive state. Absorption spectra should be compared with photoluminescence-excitation spectra, excitation–emission matrices and purified molecular controls.

## 6. Surface Passivation, Functionalization, and Doping

### 6.1. Significance of Surface Engineering

At the nanoscale, surface chemistry is part of the electronic structure of a quantum dot rather than a secondary coating. As particle size decreases, a growing fraction of atoms lies at or near the surface, where undercoordination, adsorbates, and interfacial strain can introduce states within the bandgap. This effect is especially important for silicon quantum dots (SiQDs), because even a small density of dangling bonds can open efficient non-radiative recombination pathways and sharply reduce photoluminescence [42,123]. Surface engineering must therefore be considered together with core size, crystallinity, and the intended application. Hydrogen termination, controlled oxidation, microplasma functionalization, and ligand attachment provide complementary ways to regulate surface states, colloidal stability, and chemical reactivity [24,54,124].

### 6.2. Hydrogen Passivation

Hydrogen termination is commonly used to passivate freshly prepared SiQDs. Si–H bonds can form during synthesis in hydrogen-rich plasmas or after hydrofluoric-acid etching of oxidized silicon nanocrystals [123,124]. By removing a large fraction of surface dangling bonds, hydrogen termination can produce strong band-edge photoluminescence immediately after preparation [42]. Its main limitation is poor long-term stability in air. Progressive oxidation changes the distribution of surface and interface states and may alter both the intensity and spectral position of the emission [124]. For this reason, H-terminated SiQDs are often treated as reactive intermediates for hydrosilylation or other covalent functionalization rather than as a durable final product [101].

### 6.3. Oxide and Nitride Passivation

Controlled oxidation converts the reactive SiQD surface into an amorphous SiO_2_ shell. The shell passivates dangling bonds and provides a transparent, hydrophilic, and chemically robust interface [24,123]. Loh et al. used high-pressure water-vapor annealing to prepare Si/SiO_2_ core–shell quantum dots with photoluminescence quantum yields above 40% under optimized conditions [24]. The optical response depended on annealing pressure and shell thickness, showing that oxidation cannot be treated only as protection against degradation. Interfacial strain, residual defects, and dielectric confinement also influence the emission energy and recombination dynamics.

Silicon nitride offers a complementary route when long-term environmental stability and compatibility with established silicon processing are important. Hunter et al. prepared Si/SiN_x_ core–shell nanocrystals in dual nonthermal plasma using SiH_4_, NH_3_, H_2_, and Ar [53]. After controlling oxidation, the particles reached a photoluminescence quantum yield of approximately 35% and retained improved optical performance after six months of ambient exposure. Their behavior depended on nitrogen content, shell composition, and the density of interfacial defects, which must be optimized as a coupled set of variables rather than independently.

### 6.4. Organic-Ligand Passivation and Hydrosilylation

Hydrosilylation replaces oxidation-sensitive Si–H groups with stronger Si–C bonds and provides a direct route to application-specific surface chemistry [101]. Gas-phase and solution-phase methods have been used to attach alkenes and alkynes to plasma- or chemically synthesized SiQDs [125,126,127]. The ligand layer does more than prevent aggregation. Chain length, terminal functionality, surface coverage, and steric packing affect oxidation resistance, solvent compatibility, non-radiative losses, and charge transfer across the particle-environment interface [102]. Sequential exposure to short- and long-chain unsaturated molecules can improve hydrocarbon coverage, whereas bifunctional terminal alkenes can introduce carboxyl or other polar groups for dispersion in polar media [126,127].

Biological imaging and chemical sensing generally require stable aqueous dispersions together with accessible functional groups. Suitable approaches include hydrosilylation with carboxyl-terminated alkenes, direct microplasma functionalization, PEG-containing coatings, and polymer encapsulation [54,127,128]. These modifications improve water dispersibility and provide sites for subsequent bioconjugation. Their benefits should nevertheless be evaluated against possible increases in hydrodynamic diameter, changes in cellular uptake, and losses in photoluminescence efficiency. Surface chemistry should therefore be optimized for the final biological or analytical environment rather than assessed only in the original synthesis solvent.

### 6.5. Heteroatom and Metal-Ion Doping

Heteroatom doping provides an additional means of tuning the electronic and chemical properties of carbon quantum dots (CQDs). Nitrogen, phosphorus, sulfur, and boron can be incorporated into the carbon framework or located near the surface, where they alter local charge density and introduce emissive or chemically active sites [129]. Nitrogen is the most widely studied dopant. Depending on the precursor and synthesis conditions, it may be present in pyridinic, pyrrolic, graphitic, or oxidized configurations [129,130]. These species can modify both the conjugated carbon domains and surface-state emissions. Reported improvements in photoluminescence should therefore be interpreted in relation to the bonding configuration of nitrogen, precursor chemistry, and the extent of post-synthesis purification rather than attributed to nitrogen content alone.

Sulfur-containing groups can introduce states that extend absorption toward longer wavelengths [131]. Co-doping with nitrogen and sulfur may also alter photoluminescence and sensing selectivity, but the outcome depends on the relative concentration and bonding environment of both elements [129]. As a result, elemental analysis should be combined with high-resolution X-ray photoelectron spectroscopy and fraction-resolved optical measurements when assigning a doping-induced mechanism. Metal-ion incorporation can add functionality that is not available from the carbon framework alone. Gadolinium-doped carbon dots have been investigated as dual fluorescence and magnetic-resonance probes, with Gd (III) providing paramagnetic contrast while the carbon-dot component retains optical emission [132,133]. Liao et al. reported a one-pot hydrothermal route with a longitudinal relaxivity of 14.08 mM^−1^ s^−1^ and low cytotoxicity under the conditions tested [133]. In such systems, the location and coordination state of Gd (III) are as important as the total metal content, because weakly bound ions can affect colloidal stability, toxicity, and the reliability of magnetic-resonance measurements.

### 6.6. Multilayer and Application-Oriented Surface Design

No single surface layer can meet the requirements of every application. A multilayer design can separate functions: an inner inorganic shell protects the emissive core, whereas an outer organic or polymer layer controls solubility, colloidal stability, and molecular recognition [53,128]. Amphiphilic and poly (ethylene glycol) coatings are widely used to transfer hydrophobic SiQDs into water while reducing aggregation and nonspecific interactions. Surface carboxyl groups can then be activated through carbodiimide chemistry and coupled to antibodies, peptides, or other recognition units [134].

**Figure 7:**
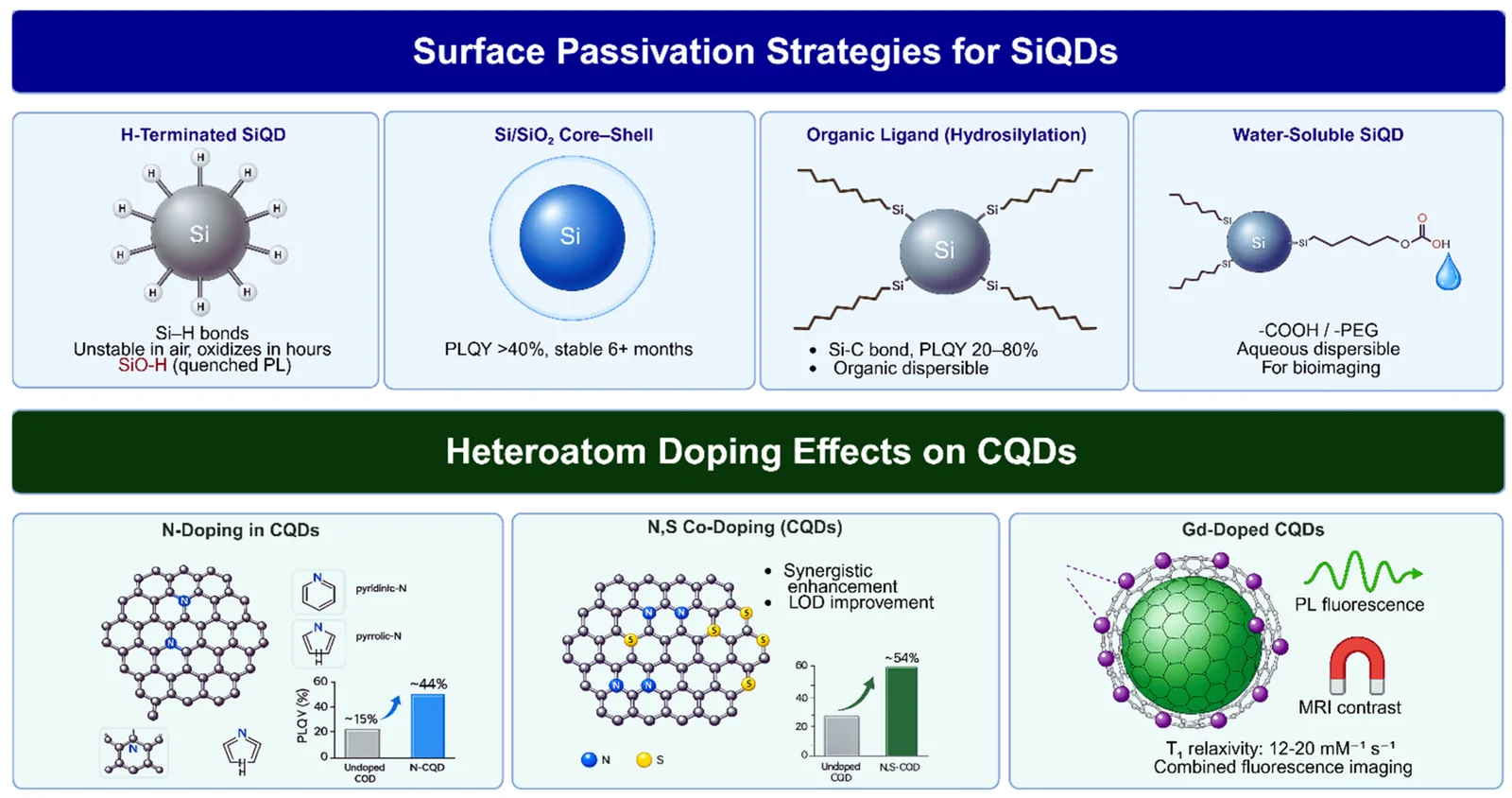
Representative surface and compositional engineering strategies for silicon and carbon quantum dots: surface passivation and functionalization of SiQDs through hydrogen termination, Si/SiO_2_ core–shell formation, hydrosilylation with organic ligands, and introduction of water-solubilizing groups; modification of CQDs through N doping, N,S co-doping, and Gd incorporation to tailor photoluminescence, sensing performance, and imaging functionality [24,53,123,124,125,126,127,128,129,131,132,133].

Each added layer changes the size and interfacial properties of the nanoparticle. Improved aqueous stability or targeting may be accompanied by a larger hydrodynamic diameter, altered cellular uptake, slower charge transfer, or reduced emission intensity. Surface engineering should therefore be evaluated using application-relevant metrics, including absolute photoluminescence quantum yield, colloidal stability, ligand density, hydrodynamic size, target specificity, and biological compatibility. Figure 7 summarizes the principal passivation and doping strategies discussed in this section.

## 7. Applications

**Figure 8:**
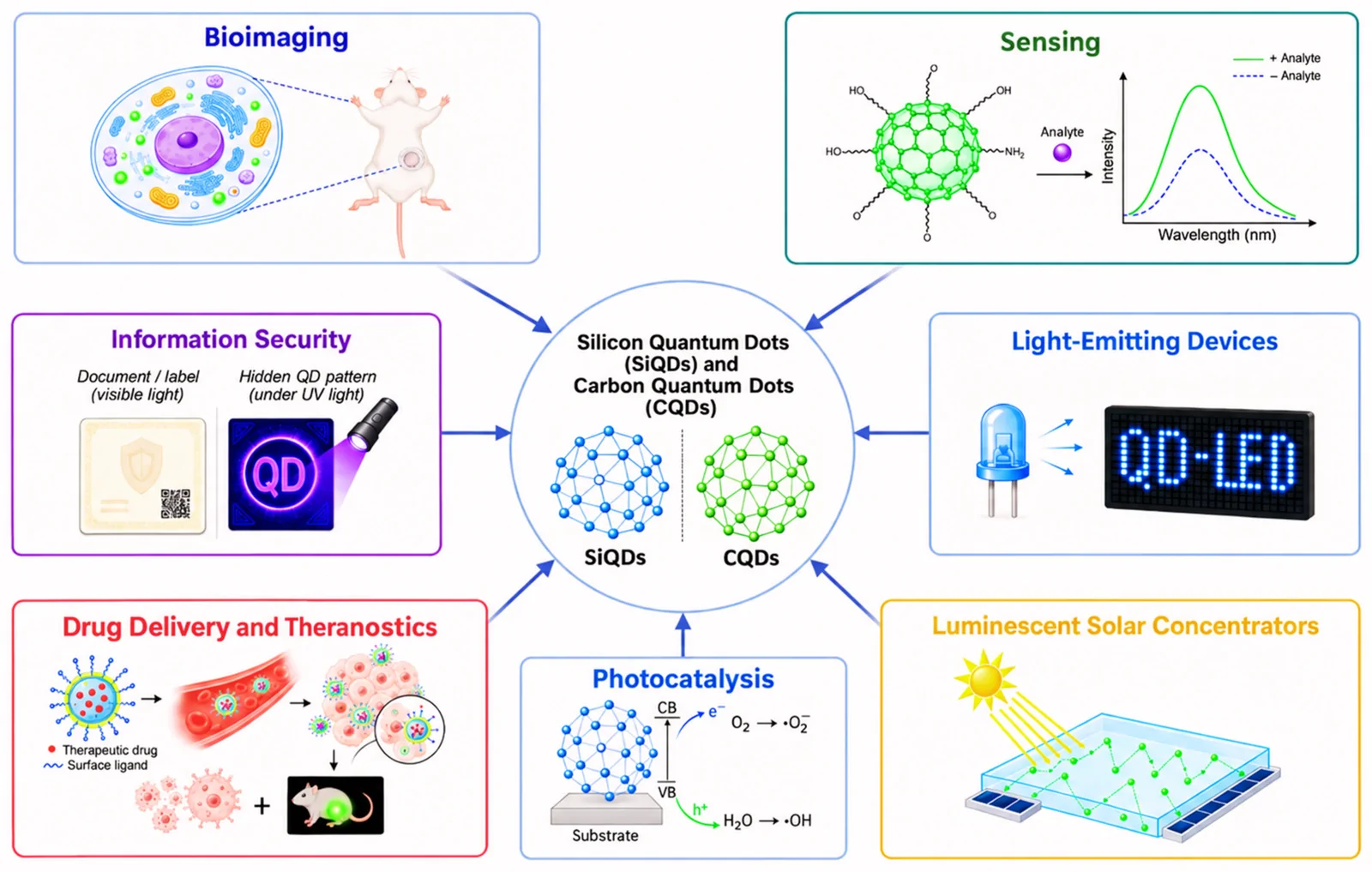
Overview of the major applications of silicon and carbon quantum dots (SiQDs and CQDs).

Only applications (Figure 8) in which material-level properties can be directly linked to a measurable device, sensing, environmental, or biological outcome are considered here. General advantages, such as the absence of heavy metals, tunable photoluminescence, and versatile surface chemistry, are therefore not reiterated in each subsection. Instead, the discussion follows a consistent framework: the structural or interfacial feature enabling a given application, the experimentally demonstrated performance metric, and the principal bottleneck limiting practical implementation. For SiQDs, these limitations commonly include surface oxidation, ligand-dependent charge transport, ensemble heterogeneity, and the preservation of surface passivation during processing. For CQDs, the measured response can be strongly influenced by purification efficiency, residual molecular fluorophores, precursor-dependent composition, and heterogeneity of surface states. In both cases, application-level performance should therefore be evaluated using the final purified and formulated material rather than relying solely on photoluminescence quantum yield (PLQY).

### 7.1. Fluorescence Bioimaging

Fluorescence bioimaging benefits from probes that combine water dispersibility, low background, photostability, and a surface that can be conjugated to targeting molecules. The first near-infrared window is commonly taken as approximately 750–1000 nm, whereas the second window extends from 1000 to 1700 nm. Relative to visible emission, these spectral regions reduce tissue scattering, absorption, and autofluorescence, with the advantage becoming more pronounced in the NIR-II range [135]. Most reported SiQD bioimaging systems still emit in the red or NIR-I region rather than NIR-II, but they offer a second form of contrast: microsecond-scale emission can be separated temporally from the nanosecond autofluorescence of cells and organic dyes. Recent reviews of functionalized SiQD nanoagents highlight surface oxidation, ligand grafting, polymer coating, and biomolecular conjugation as the main routes to aqueous stability and biological targeting [88]. Tu et al. demonstrated this lifetime advantage directly using antibody-conjugated SiQD nanoparticles with a photoluminescence lifetime of about 25 microseconds. Time-gated confocal imaging separated the long-lived SiQD signal from endogenous cellular luminescence and spectrally overlapping organic dyes, enabling multiplexed imaging of live cancer cells [136]. This result is more specific than a general claim of improved brightness: the main gain came from temporal discrimination rather than from a higher steady-state fluorescence intensity. Plasma synthesis can couple core formation with surface conversion. Lee et al. introduced oxygen, water vapor, and acrylic acid into the afterglow of nonthermal plasma to obtain water-dispersible SiQDs. After aqueous aging for eight days, the particles emitted near 830 nm and reached a PLQY of approximately 30% [88]. The time-dependent increase in PLQY is relevant for biological use because it shows that storage history and continuing surface passivation must be reported together with the nominal particle formulation. CQDs provide a complementary platform because their hydrophilic surface groups and low-cost precursors simplify dispersion and conjugation. Atchudan et al. prepared approximately 5 nm CQDs from banana-peel waste with a reported PLQY of 20% and demonstrated fluorescence imaging in nematodes at biocompatible concentrations [28]. CQDs can also be combined with magnetic ions to add an anatomical imaging modality. Pan et al. synthesized Gd-doped CQDs with an absolute fluorescence quantum yield of 40% and a longitudinal relaxivity of 57.42 mM^−1^ s^−1^ and demonstrated fluorescence/magnetic-resonance imaging in cells and mice [137]. Such systems improve multimodal capability but introduce an additional safety question: total gadolinium content, ion leakage, renal clearance, and long-term retention must be established rather than inferred from the nominal biocompatibility of the carbon matrix. These studies show that bioimaging performance is governed by the final purified formulation rather than by the nominal material class alone. For SiQDs, oxidation/passivation history controls the lifetime and red/NIR emission that enable time-gated detection; for CQDs, residual fluorophores, dopants, surface charge, and purification can strongly influence both brightness and biological response. Translation therefore requires reporting of purified particle dose, hydrodynamic size in biological media, serum stability, protein-corona formation, biodistribution, degradation products, and clearance over time.

### 7.2. Chemical Sensing and Biosensing

SiQD- and CQD-based sensors commonly operate through fluorescence quenching, fluorescence enhancement, or a ratiometric change between two bands. The observed response can result from photoinduced electron transfer, resonance energy transfer, static complex formation, analyte-induced aggregation, or inner-filter effects. A calibration curve alone does not distinguish between these mechanisms. Absorption overlaps, fluorescence lifetime, dilution controls, pH dependence, and competitive-analyte tests should be evaluated before a signal change is assigned to a specific surface interaction. Surface-functionalized SiQDs produced by a microplasma electrochemical process have been used for label-free dopamine detection. Chang et al. reported a linear range of 0.83–83.33 micromolar and a detection limit of 0.32 micromolar, with the response attributed to surface interactions and fluorescence-resonance energy transfer [54]. The practical advantage of this route is the direct formation of colloidally stable, amine-containing particles; the limitation is that selectivity and response can depend strongly on the ionic composition and pH of the sample matrix. Biomass-derived CQDs cover a wider range of analytes but are more sensitive to feedstock composition and purification. Water-hyacinth CQDs detected Fe^3+^ with a reported limit of detection of 0.084 micromolar and were also used for fluorescence imaging of *Klebsiella* cells [60]. N, S-containing CQDs from pomelo peel supported a turn-off-on assay with detection limits of 0.086 micromolar for Fe^3+^ and 0.34 micromolar for L-cysteine [103]. These values should not be ranked without considering the buffer, linear range, blank variation, competing ions, recovery in real samples, and the statistical definition of the detection limit. For CQDs, chromatographic purification is important because residual molecular fluorophores may respond to the analyte independently of the nanoparticle fraction.

### 7.3. Light-Emitting Diodes

In light-emitting devices, the relevant question is not solution PLQY alone but whether the emissive layer preserves radiative efficiency while supporting charge injection and transport. For SiQDs, surface ligands that suppress nonradiative defects can simultaneously increase electrical resistance; for CQDs, structural heterogeneity and molecular emitters can complicate reproducible solid-state electroluminescence. SiQDs are attractive as heavy-metal-free red emitters because band-edge emission can be efficient and relatively stable, but their long organic ligands often hinder carrier transport. Ueda and Saitow reported red-emitting SiQDs with PLQYs of 60–80% and LEDs with external quantum efficiencies above 10% using a low-cost hydrogen-silsesquioxane precursor route [40]. Xu et al. showed why particle brightness and device efficiency must be evaluated separately: changing decyl-ligand coverage had only a modest effect on PL peak position and PLQY yet produced large changes in turn-on voltage and electroluminescence efficiency because dense ligand shells electrically isolated the dots [56]. Surface passivation and electronic coupling therefore require a deliberate compromise rather than independent optimization. CQDs can function as color-conversion phosphors or as the electroluminescent semiconductor itself. Yuan et al. reported hexagonal CQDs with a PLQY of 96%, electron mobility of 0.07 cm^2^ V^−1^ s^−1^, and electroluminescent devices with an external quantum efficiency of 4.6% and brightness above 11,000 cd m^−2^ [138]. This performance illustrates the potential of structurally defined CQDs, but it should not be generalized as heterogeneous biomass-derived dots. The high PLQY values reported for the SiQD and CQD systems discussed above originate from different material classes, sample preparations, and measurement contexts and are therefore cited here to illustrate achievable optical performance rather than to establish a numerical ranking between SiQDs and CQDs. For both SiQDs and CQDs, device studies should report film PLQY, charge mobility, turn-on voltage, spectral stability, operational lifetime, and batch-to-batch variation alongside the solution properties of the starting colloid.

### 7.4. Luminescent Solar Concentrators

For luminescent solar concentrators, the decisive materials problem is maintaining high emission while minimizing reabsorption, scattering, and long-term optical loss as the waveguide area increases.

SiQDs are promising LSC luminophores because they combine broad absorption, red/NIR emission, elemental abundance, and potentially large Stokes shifts. Surface stability remains decisive. Loh et al. used high-pressure water-vapor annealing to form Si/SiO_2_ core–shell QDs with environmentally stable PLQYs above 40%, providing a rapid inorganic passivation route relevant to solar concentrators and other solid-state devices [24]. However, particle PLQY alone does not establish LSC efficiency; waveguide transparency, loading-dependent scattering, edge coupling, and outdoor stability must be measured at device scale.

Carbon dots can also achieve strong spectral separation. Li et al. reported yellow-emitting CDs with a 193 nm Stokes shift and a quantum yield of 61%. A laminated 10 × 10 cm^2^ LSC reached an optical conversion efficiency of 4.56% and a power-conversion efficiency of 4.1% under natural sunlight [139]. This study demonstrates that suppression of reabsorption can compensate for the broader structural heterogeneity of carbon dots. Direct comparisons between SiQD- and CQD-based LSCs should nevertheless use the same device area, geometric gain, illumination spectrum, edge-cell configuration, and aging protocol.

### 7.5. Photocatalysis and Environmental Remediation

Carbon-based quantum dots can serve as photosensitizers, electron-transfer mediators, or co-catalysts in semiconductor composites. Their contribution may include extension of visible-light absorption, interfacial charge separation, adsorption of reactants, and transfer of photogenerated carriers. These roles depend on the band alignment and surface chemistry of the complete composite; the presence of fluorescent dots does not by itself prove photocatalytic charge transfer. Time-resolved spectroscopic techniques, particularly transient absorption spectroscopy and TRPL, are therefore valuable for resolving charge separation, trapping, migration, and interfacial transfer kinetics in QD-based photocatalytic systems [140]. Recent reviews describe applications in pollutant degradation, hydrogen evolution, carbon-dioxide reduction, and nitrogen conversion [141]. Beyond photocatalysis, quantum dots have also been incorporated into nanofiltration membranes, where their tunable surface chemistry can influence permeability, antifouling behavior, and ion selectivity [142]. Photocatalytic performance should be reported with photon flux, catalyst loading, dark adsorption, dissolved oxygen, pH, initial pollutant concentration, mineralization or total-organic-carbon removal, intermediate products, and catalyst recovery. Apparent dye disappearance can otherwise reflect adsorption, sensitized photobleaching, or spectral interference rather than complete degradation. Circular synthesis strategies offer a separate environmental benefit. Chen et al. converted the pollutant Reactive Red 2 into fluorescent carbon nanodots and demonstrated reduced toxicity and bioimaging in cells and zebrafish [109]. That work supports waste valorization, but it should not be cited as direct evidence of photocatalytic degradation because photocatalysis was not the main demonstrated application.

### 7.6. Drug Delivery and Theranostics

The same functional groups that support sensing and bioimaging can be used to bind drugs through electrostatic interactions, hydrogen bonding, covalent coupling, or pi–pi stacking. CQDs are currently more widely explored than SiQDs for this purpose because their surface chemistry is easier to tune in water. The main advantage is the possibility of tracking the carrier by fluorescence; the main risk is that fluorescence can persist after the therapeutic cargo has been released or after the carrier has chemically changed. Mathew et al. developed a chitosan/carbon-dot matrix for dopamine delivery. The composite showed approximately 97% cell viability in the tested cell lines, encapsulation efficiency above 80%, and sustained in vitro release, while the carbon-dot emission enabled optical tracking [143]. More complex therapeutic roles have also been demonstrated. Mannose-derived carbon dots were used to capture danger signals generated by microwave ablation and deliver them to dendritic cells, thereby enhancing antitumor immune responses in vivo [144]. In another strategy, hematoporphyrin was incorporated into CQDs in a one-step microwave synthesis; the resulting water-soluble particles generated reactive oxygen species under deep-red illumination and showed photodynamic activity against MCF-7 cells [145]. These examples illustrate different functions–carrier, immunoadjuvant, and photosensitizer and should not be grouped under a single claim of drug delivery. Translation requires separate measurements of loading capacity, release kinetics, therapeutic dose, dark toxicity, phototoxicity, pharmacokinetics, off-target accumulation, and degradation. SiQD-based delivery remains less developed and will require evidence that surface oxidation and biodegradation do not produce harmful or poorly cleared intermediates.

### 7.7. Information Security and Anti-Counterfeiting

Carbon dots are well suited to security inks because their emission can be tuned by excitation wavelength, composition, aggregation state, or delayed phosphorescence. Multilevel authentication can be created by combining prompt fluorescence with a second response that appears only after a stimulus or after the excitation source is removed. Jiang et al. converted fluorescent polymer carbon dots into an ultralong room-temperature phosphorescent material by heating. The afterglow lasted for about 10 s, with a phosphorescence lifetime of 1.39 s, and the aqueous dispersion was demonstrated as an ink for information encryption and anti-counterfeiting [146]. For practical use, optical complexity is only one requirement. Security patterns must also withstand humidity, solvents, abrasion, repeated bending, and prolonged illumination, while remaining compatible with high-throughput printing and inexpensive excitation sources. The use of chemically heterogeneous CQDs can increase coding diversity but may also complicate color consistency and long-term reproducibility.

## 8. Challenges and Future Perspectives

### 8.1. Scalability and Manufacturing

Moving from laboratory synthesis to reproducible continuous production remains a central engineering challenge (Table 3) for both silicon quantum dots (SiQDs) and carbon quantum dots (CQDs). Plasma reactors are attractive because nucleation and growth occur on short time scales and can be tuned through RF power, gas composition, pressure, and residence time. The atmospheric-pressure HelixJet has demonstrated that crystalline, photoluminescent silicon nanocrystals can be produced without a conventional low-pressure reactor, while retaining control over particle size through the operating conditions [49]. More recent plasma-engineering work has further shown that surface treatment and size control can be combined to obtain bright SiQDs with near-unity internal quantum yield in the emissive fraction [25]. These advances are important, but scale-up cannot be reduced to increasing the reactor volume. Collection efficiency, precursor utilization, residence-time distribution, downstream passivation, and optical uniformity must all be maintained as throughput increases. For plasma reactors, scale-up claims should therefore include a mass and energy balance-precursor conversion, recovered dry-product yield, collection efficiency, specific energy use, and long-duration operating stability-together with the optical and structural quality of the recovered fraction.

For CQDs, continuous hydrothermal flow processing offers a practical alternative to batch autoclaves. Continuous-flow synthesis has been demonstrated with targeted biomass-derived precursors [147] and with simple sugars in a pressurized microreactor [148]. In the latter study, sucrose-derived carbon dots were produced at 6.6 mg min^−1^ with a reported quantum yield of 53.03% under the optimized flow conditions [148]. These results show that continuous production is feasible, but throughput alone is not an adequate measure of scalability. Future studies should report purified mass yield, precursor conversion, energy demand, purification losses, and product quality alongside PLQY.

### 8.2. Long-Term Stability

For SiQDs, long-term optical performance is often governed by changes at the surface rather than by degradation of the crystalline core. Oxidation, ligand loss, aggregation, and changes in the local dielectric environment can alter both emission intensity and spectral position. Plasma-induced acrylic-acid grafting followed by PEGylation has produced Si nanocrystals that remain dispersible in water and high-ionic-strength media, with near-infrared photoluminescence retained after transfer to aqueous conditions [149]. A later single-step plasma process integrated oxidation and acrylic-acid grafting in the afterglow and produced water-soluble SiQDs whose PLQY increased during aging in water, reaching approximately 30% after eight days [88]. These observations are encouraging, but they also show that the optical properties of SiQDs can continue to evolve after synthesis. Stability should therefore be evaluated under prolonged illumination, storage, temperature cycling, and the chemical conditions relevant to the intended application.

CQDs are often compatible with aqueous media, but their fluorescence can still change with ionic environment, pH, oxidation, aggregation, and residual molecular species. Protein-derived carbon dots, for example, have shown strong colloidal stability in concentrated salt solutions [150]. Such results should not be generalized across all CQD formulations. Stability measurements are most useful when they are performed in the same medium, concentration range, and time window expected during practical use.

### 8.3. Toxicological Assessment and Regulatory Translation

The absence of cadmium or lead is an important advantage of SiQDs and CQDs, but it is not sufficient to establish biological safety. Nanoparticle response depends on the complete formulation, including size, surface charge, ligand chemistry, aggregation state, dose, impurities, and exposure time [151]. Water-soluble plasma-synthesized SiQDs have shown encouraging short-term cell-viability results [88], while continuously produced sugar-derived CQDs were well tolerated by L-929 fibroblast cells over the concentration range examined in the original study [148]. These findings are useful for screening, but they do not replace studies of biodistribution, degradation, clearance, immune response, and chronic exposure.

For biomedical translation, toxicological testing should be tied to the same purified particle fraction that is used for optical characterization and application studies. Residual precursors, purification by-products, surface coatings, and chemical changes during storage or exposure to biological fluids should be reported explicitly. A formulation-specific safety assessment is more informative than assigning a general biocompatibility label to an entire class of quantum dots.

### 8.4. Photoluminescence Efficiency: From Record Values to Reproducibility

Recent studies show how far SiQD optical performance has progressed. Plasma-engineered samples have reached near-unity internal quantum yield for the emitting population [25], and size-separated plasma-grown silicon nanocrystals have achieved ensemble PLQYs approaching 70% [39]. Continuous-flow CQD synthesis has also produced materials with a reported quantum yield above 50% under optimized conditions [148]. These values are not directly interchangeable. Internal quantum yield, ensemble PLQY, absolute PLQY, and device external quantum efficiency describe different quantities and should be identified clearly whenever results are compared.

The next challenge is reproducibility rather than another isolated record value. High PLQY should be demonstrated across independent syntheses and retained after purification, ligand exchange, film formation, or transfer into the final device or biological medium. Reporting the fraction of optically dark, aggregated, or discarded material would also make high efficiency claims easier to interpret. Common measurement protocols and uncertainty estimates would substantially improve comparison between synthesis routes.

### 8.5. Mechanistic Understanding and Data-Guided Optimization

The remaining mechanistic questions are different for SiQDs and CQDs. In crystalline SiQDs, quantum confinement provides the basic framework, but the measured emission also reflects surface chemistry, oxidation, and the competition between phonon-assisted and faster quasi-direct recombination channels [25,39]. In CQDs, the emitting species may reside in the carbonized particle, at surface or defect sites, or in molecular fluorophores generated during synthesis. Separation and chemical identification of low-molecular-weight species have shown that these fluorophores can contribute substantially to the observed fluorescence [152].

Progress therefore depends on linking structure and optical dynamics within the same purified fraction. Single-particle spectroscopy, time-resolved measurements, controlled fractionation, and surface-sensitive analysis are particularly valuable for this purpose. Atomistic modelling can help test plausible surface configurations, while data-driven optimization could be used to explore the large parameter space of plasma power, gas composition, pressure, residence time, and post-treatment. For plasma synthesis, however, closed-loop optimization should still be regarded as a developing research direction rather than an established production strategy.

### 8.6. Hybrid Synthesis Strategies

Hybrid processing offers a practical way to combine the strengths of gas-phase plasma synthesis with solution-phase surface chemistry. A plasma can form crystalline silicon cores rapidly, after which solution-phase or polymer-based treatment can provide aqueous dispersibility, bioconjugation sites, or application-specific surface groups. Plasma-induced acrylic-acid grafting followed by PEGylation is one example of this sequential strategy [149]. More recently, oxidation and ligand grafting were incorporated directly into the plasma afterglow, allowing water-soluble NIR-emitting SiQDs to be produced in a single processing sequence [88].

These studies suggest that particle formation and surface engineering should be designed together rather than optimized as independent steps. Modular plasma/afterglow/liquid configurations are particularly attractive because they can separate control of the crystalline core from control of the final interface while minimizing unnecessary transfer and purification steps.

### 8.7. Emerging Applications

The application landscape is expanding beyond conventional fluorescence imaging and chemical sensing. Silicon QDs have been incorporated into light-emitting devices operating at the lower edge of the NIR-II spectral window; a colloidal Si-QLED with electroluminescence near 1.0 μm and an external quantum efficiency of 4.84% has been demonstrated [153]. Water-soluble NIR-emitting SiQDs also provide a heavy-metal-free platform for optical bioimaging [88]. CQDs remain attractive for applications that benefit from solution processability and chemically diverse surfaces, including imaging, photocatalysis, and sensing [61].

The most compelling applications are likely to be those that exploit a material-specific advantage rather than simply replacing an established quantum dot. For SiQDs, this may mean combining high-purity crystalline cores with long-wavelength emission in optoelectronic or photonic devices. For CQDs, low-cost precursor chemistry and adaptable surface functionality may be more valuable in sensing, interfacial chemistry, and disposable or large-area systems.

### 8.8. Sustainability and Circular-Economy Considerations

The environmental value of a synthesis route should be judged from the complete process rather than from the origin of the precursor alone. Biomass- and sugar-derived CQDs can reduce reliance on petrochemical feedstock, and continuous hydrothermal processing can shorten reaction times and increase productivity [147,148]. At the same time, heating, water and solvent consumption, purification, low product yield, and waste generation can offset part of this advantage.

The same reasoning applies to plasma synthesis. Gas-phase processing can reduce solvent use and provide rapid particle formation, but electrical power, precursor efficiency, vacuum operation where required, and nanoparticle collection must be included in any meaningful comparison [25,49]. Future studies would benefit from cradle-to-gate life-cycle assessment combined with transparent reporting of purified material yield, energy use, and waste streams. The use of silicon- and carbon-rich waste as feedstocks is promising, but its environmental benefit should be demonstrated quantitatively rather than assumed.

## 9. Conclusions

Plasma-assisted and green solution-phase synthesis have brought SiQDs and CQDs to a stage where the main challenge is no longer simply producing bright nanoscale emitters, but controlling what is produced, reproducing it across batches, and retaining its properties during application-specific processing. The two material families reach this point through fundamentally different structure–property relationships.

For SiQDs, plasma and related gas-phase methods provide unusually direct control over nanocrystal formation, crystallinity, charging, and surface termination. High internal radiative efficiencies demonstrate the optical potential of well-passivated Si cores, but technologically relevant performance will depend on ensemble PLQY, collection efficiency, downstream passivation, and the ability to maintain narrow and reproducible particle populations during continuous production.

Green synthesis of CQDs has expanded well beyond conventional hydrothermal carbonization. Microwave-assisted processing, biomass conversion, plant-derived precursors, and electrochemical methods can produce fluorescent CQDs with heteroatom-rich surfaces and application-specific functionality [37,46,60,61,96,154]. The chemical diversity of natural precursors can support self-doping and reduce the need for separate functionalization steps. At the same time, variation in precursor composition remains a major source of batch-to-batch inconsistency. Better control of feedstock quality, purification, and carbonization pathways is therefore required before these methods can be considered genuinely reproducible and scalable.

The principal value of comparing SiQDs and CQDs is not that they share a common photophysical mechanism, but that their differences reveal which synthesis descriptors are genuinely transferable. In crystalline SiQDs, particle size, core crystallinity, surface termination, oxidation, and interface quality can be related to a defined semiconductor core. In CQDs, the microscopy-defined particle diameter may not correspond to the dominant emissive unit because carbon domains, surface- or defect-related states, polymer-like structures, and molecular fluorophores can coexist. Consequently, identical terms such as “PLQY,” “surface passivation,” or “size control” can describe physically different situations. A critical comparison must therefore follow the complete chain from precursor activation and particle formation to purification, surface chemistry, photophysics, and the final application-specific formulation [3,9,13,27,30,35,43,55,74,84,92].

The application range of SiQDs and CQDs now extends beyond bioimaging, sensing, and LEDs to luminescent solar concentrators, photocatalysis, theranostics, drug delivery, anti-counterfeiting, wearable sensors, and quantum photonics. Their low elemental toxicity and reliance on earth-abundant components are important advantages, but neither should material family be regarded as universally safe. Toxicological behaviour depends on particle size, dose, surface charge, precursor residues, and exposure route, and must be evaluated for each formulation.

Within this framework, low-pressure nonthermal plasma offers particularly strong control over gas-phase SiQD nucleation, growth, charging, crystallinity, and surface processing, whereas solution-phase and green routes provide broader chemical flexibility and are especially powerful for CQD precursor conversion and functionalization. Hybrid plasma–liquid and sequential plasma/solution-phase strategies may potentially combine these strengths, but their practical value will depend on reproducible product identity rather than on record optical values alone. Future progress should therefore prioritize standardized PLQY reporting, fraction-resolved characterization, precursor and particle mass balances, collection efficiency, specific energy consumption, long-duration reactor stability, and independent batch statistics. These common criteria constitute the central cross-material contribution of this review and provide a basis for designing scalable, mechanistically interpretable, and application-ready Si- and C-based quantum dots.

## Figures and Tables

**Table 1:** Structural classification and identification of Si- and C-based quantum dots.

QD Class	Structural Description	Typical Dimensions	Key Surface/ Compositional Features	Dominant/Possible PL Origin	Required Characterization	Main Interpretative Limitation	Ref
**Crystalline SiQDs**	Diamond–cubic Si nanocrystalline core	typically ~1–10 nm; representative luminescent examples ~1.5–5.5 nm	H termination, oxide, organic ligands	Quantum-confined Si-core states modified by surface/interface states	HRTEM, SAED, XRD, XPS, TRPL	Emission cannot be determined by size; oxidation, strain and defects alter PL	[39,40,49,50]
**Amorphous/partially crystalline SiQDs**	Short-range tetrahedral Si without full long-range order	typically a few nm (<10 nm); no universal class-specific range	H incorporation, structural disorder, defects	Disorder/localized states plus confinement effects	HRTEM, diffraction, Raman/XPS, H-content analysis	Absence of resolved lattice fringes alone does not establish a fully amorphous structure	[22,41,50,51,52]
**Si/SiO_2_ core–shell QDs**	Si-rich/crystalline core with silica shell	Si core ~1–5 nm; total diameter depends on oxide-shell thickness	Controlled oxide shell	Si-core emission strongly modified by interface/passivation	TEM, XPS, oxide thickness, PL/TRPL	Shell thickness and interface quality influence PL; oxide is not merely an inert coating	[24]
**Si/SiN_x_ core–shell QDs**	Si core surrounded by nitride-rich layer	Si core usually <~5 nm for quantum-confined PL; total size shell-dependent (reported ~5–10 nm)	Nitride passivation; possible subsequent oxidation	Core-related emission with surface/interface contribution	TEM, XPS, N-content, PL	Incomplete coverage, strain and interfacial traps can offset passivation	[53]
**Matrix-embedded SiQDs**	Si nanocrystals dispersed within SiO_2_, SiN_x_, SiC or related film	~2–10 nm Si nanocrystals; strongly matrix- and annealing-dependent	Continuous solid host	Si-core + matrix/interface-related emission	TEM/XRD, Raman, XPS, film spectroscopy	PL cannot be assigned to the Si core because matrix defects may emit	[51,52]
**Surface-functionalized SiQDs**	Colloidal SiQDs with covalently bound organic/polymeric surface groups	Si core ~1–5 nm (up to ~8 nm reported); hydrodynamic size ligand-dependent	Alkyl, carboxyl, amine, PEG-type ligands	Core and/or surface-influenced emission	XPS/FTIR, ligand density, hydrodynamic size, PL/TRPL	Ligand length alone does not predict PLQY or device performance	[6,54,55,56]
**Graphitic CQDs**	Compact carbon nanoparticles containing sp^2^-rich domains	~2–10 nm	O/N-containing edge and surface groups	π-conjugated domains plus edge/surface/defect states	HRTEM, Raman, XPS, XRD, spectroscopy	Excitation-dependent PL is not proof of particle-size dispersion	[3,43,44]
**Carbonized polymer dots (CPDs)**	Cross-linked polymer-like matrix with partially carbonized domains	typically ~1–10 nm; occasional >10 nm reports	Polymer-derived functional groups and fluorophores	Molecular fluorophores, carbonized domains, crosslink-enhanced emission	Chromatography/fractionation, spectroscopy, TEM, XPS	Structurally heterogeneous; high PLQY is not a defining class property	[57]
**Graphene quantum dots (GQDs)**	Finite single- or few-layer graphene fragments	commonly <10 nm lateral size; ~0.4–4 nm thickness	Edge groups, oxidation, layer-number effects	Lateral confinement and edge/surface states	AFM/TEM, Raman, XPS, spectroscopy	Should not be conflated with quasi-spherical graphitic CQDs	[3,58,59]
**Heteroatom-modified CQDs**	Any CQD architecture modified with N, S, P, B, etc.	typically <10 nm; no class-specific range (depends on parent CQD architecture)	Pyridinic, pyrrolic, graphitic N; S/P/B species, etc.	Modification of core/surface electronic states	High-resolution XPS + elemental analysis + PL	Dopant identity or atomic % alone does not predict emission wavelength or PLQY	[45,46,47,48]
**Biomass-derived CQDs**	Precursor-defined family rather than unique structural class	commonly ~2–10 nm; precursor- and process-dependent	O-rich surface; possible N/S depending on precursor	Core, surface, polymeric and/or molecular contributions	XPS, elemental analysis, chromatography, TEM, PL	Feedstock variability and residual molecular fluorophores complicate comparison	[27,28,45,60,61,62,63]
**SiC QDs**	Covalently bonded Si–C nanocrystals, cubic or hexagonal polytypes	~2–4 nm in cited plasma example	Oxidized surface possible	Size- and surface-dependent; PL not universal	HRTEM/XRD, composition, surface analysis	Some plasma-produced SiC nanocrystals show no measurable PL	[64,65]

**Table 2:** Photoluminescence mechanisms and minimum evidence required for assignment.

Proposed Emissive Mechanism	Material	Typical Time Scale Described in Manuscript	Physical/Chemical Origin	Observations Consistent with Mechanism	Evidence That Is Insufficient by Itself	Stronger Verification Strategy
Quantum-confined crystalline-core emission	SiQDs	typically tens to hundreds of μs; μs-ms depending on core size/surface chemistry	Recombination of carriers confined in crystalline Si core	Size-dependent optical gap/emission; long-lived red/NIR PL	PL wavelength alone	Size-fractionated TEM/HRTEM + TRPL + surface analysis
Quasi-direct/enhanced zero-phonon core contribution	Ultrasmall/strongly confined SiQDs	sub-ns to ns fast components; slower μs-scale core PL may coexist	Strong confinement and interface-induced intervalley mixing that enhance nominally zero-phonon transitions	Fast zero-phonon contribution increases as nanocrystal size decreases; ultrafast high-energy PL components	Short lifetime alone	Size-fractionated structural analysis + ultrafast TRPL + absorption + atomistic modelling
Surface/interface-related emission	SiQDs	variable	Oxidation-, interface-, defect- and ligand-induced electronic states; some dangling bonds act as nonradiative traps	PL changes with oxidation/passivation	Size-dependent PL alone	XPS/FTIR ± EPR + controlled surface modification/oxidation + TRPL + temperature-dependent PL
π-conjugated domain emission	CQDs	typically ns-scale fluorescence	Quantum-confined, conjugation-length-dependent electronic states in sp^2^-rich carbon domains	Spectral changes correlated with carbonization/core structure	TEM particle diameter	Raman/XPS + purification + excitation–emission mapping
Surface/defect state emission	CQDs	ns-scale; heterogeneous/multi-exponential	Functional groups, defect-localized electronic states	Excitation-dependent PL; chemistry-dependent emission	Excitation-dependent PL alone	Surface-sensitive analysis + TRPL + fraction-resolved PL
Molecular-fluorophore emission	CQDs/CPDs	typically molecular ns-scale fluorescence	Molecular products generated during bottom-up synthesis	Strong PL in low-molecular-mass fractions; chromatography-dependent emission spectra	TEM confirmation of nanoparticles in same crude sample	HPLC/SEC/electrophoresis + MS/NMR where appropriate + PL of separated fractions
Crosslink-enhanced emission	CPDs	variable	Restriction of nonradiative molecular motion and rigidification of sub-fluorophoric units in a crosslinked polymer network	Enhanced fluorescence and/or delayed emission upon increased crosslinking or rigidification	High PLQY alone	Structural comparison across degree of crosslinking + spectroscopy
Mixed emissive states	CQDs	typically multi-exponential ns-scale decay; slower components may occur	Carbon-core + surface + molecular centers	Multi-exponential lifetime; excitation-dependent spectral changes	Any single steady-state PL spectrum	EEM + TRPL + chromatography + chemical identification

**Table 3:** Critical bottlenecks and research roadmap.

Challenge	SiQD-Specific Problem	CQD-Specific Problem	What Should Minimally Be Reported	Recommended Research Direction
Reproducible scale-up	Maintaining size, crystallinity, residence time and downstream passivation at higher throughput	Maintaining precursor composition, carbonization and purification consistency	Purified/recovered mass yield; precursor feed and conversion/utilization; collection efficiency; throughput; residence time; specific energy demand; discarded fraction; continuous operating time; batch/run statistics	Continuous reactors + in-line/operando diagnostics
Optical reproducibility	Ensemble PLQY can remain far below internal QY because of dark/defective particles	Highly emissive molecular fluorophores may dominate crude-sample PL	Absolute PLQY, excitation wavelength, concentration, uncertainty, ≥independent batches, purified fraction identity	Standardized PLQY protocols + fraction-resolved measurements
Structural assignment	Core size vs oxide/interface contribution	TEM nanoparticle size may not equal emissive-domain size	HRTEM/XRD + XPS + TRPL; chromatography for CQDs	Multimodal characterization of the exact same fraction
Long-term stability	Oxidation, ligand loss, aggregation, dielectric-environment changes	pH, ionic strength, oxidation, aggregation, residual molecular species	Storage time, atmosphere, illumination dose, medium, temperature, before/after PLQY	Accelerated and application-specific aging protocols
Surface engineering	Passivation can improve PL while reducing charge transfer/transport	Functional groups improve sensing but increase chemical heterogeneity	Ligand identity/density, hydrodynamic size, surface charge, oxidation state	Application-driven optimization instead of maximizing PLQY alone
Toxicological translation	Biological response depends on ligand/surface oxide and degradation products	Residual molecules, precursor-derived impurities and heteroatom/metal species may dominate response	Purified dose, impurities, surface charge, aggregation, exposure time, biodistribution/clearance	Formulation-specific toxicity studies on same fraction used for optics
Sustainability	Electricity, precursor utilization, vacuum operation, particle collection	Heating, solvent/water use, dialysis, low yield, feedstock pretreatment	Energy per mass product, water/solvent use, purified yield, waste streams	Cradle-to-gate life-cycle assessment
Biomass reproducibility	Usually not applicable to conventional SiQD synthesis; for biomass-/waste-derived Si precursors, variability in SiO_2_ content, mineral impurities and pretreatment can affect Si recovery and final SiQD properties	Species, season, storage and composition alter C/N ratio and fluorophore formation	Moisture, ash, elemental composition, extraction conditions, solid/liquid ratio	Standardized feedstock characterization and reference-model precursors
Mechanistic understanding	Relative role of quantum-confined core, interfaces and quasi-direct channels	Core states vs defects vs molecular fluorophores	Fractionation + time-resolved spectroscopy + chemical identification	Single-particle/fraction-resolved spectroscopy + modelling
Hybrid processing	Integrating core synthesis with oxidation/passivation without transfer losses	Controlling interfacial plasma chemistry without producing undefined molecular emitters	Stage-resolved structure/property data	Modular plasma → afterglow → liquid processing
Data-driven optimization	Large parameter space in power, gas chemistry, pressure and residence time	Large parameter space in precursor ratio, pH, T/time and purification	Complete process metadata and uncertainty	Closed-loop/ML-guided optimization after standardized datasets become available

## Data Availability

No new data were created or analyzed in this study. Data sharing is not applicable to this article.

## References

[1] Canham L.T. (1990). Silicon Quantum Wire Array Fabrication by Electrochemical and Chemical Dissolution of Wafers. Appl. Phys. Lett..

[2] Xu X., Ray R., Gu Y., Ploehn H.J., Gearheart L., Raker K., Scrivens W.A. (2004). Electrophoretic Analysis and Purification of Fluorescent Single-Walled Carbon Nanotube Fragments. J. Am. Chem. Soc..

[3] Wang B., Lu S. (2022). The Light of Carbon Dots: From Mechanism to Applications. Matter.

[4] Zhang L., Zhou H., Chen Y., Zheng Z., Huang L., Wang C., Dong K., Hu Z., Ke W., Fang G. (2024). Spontaneous Crystallization of Strongly Confined CsSnxPb1-XI3 Perovskite Colloidal Quantum Dots at Room Temperature. Nat. Commun..

[5] Zhang L., Chen Y., Zheng Z., Zhou Y., Li C., Li G., Ren B., Hu Z., Zhou H., Ren F. (2025). Rapid Room Temperature Entropy-Stabilized Synthesis Enabling Super-Stable Metal Halide Perovskite Semiconductor Colloidal Nanocrystals. Adv. Funct. Mater..

[6] Yang D., Cui Z., Wen Z., Piao Z., He H., Wei X., Wang L., Mei S., Zhang W., Guo R. (2023). Recent Updates on Functionalized Silicon Quantum-Dot-Based Nanoagents for Biomedical Applications. ACS Mater. Lett..

[7] Zhang L., Yang X., Yin Z., Sun L. (2022). A Review on Carbon Quantum Dots: Synthesis, Photoluminescence Mechanisms and Applications. Luminescence.

[8] Iravani S., Varma R.S. (2020). Green Synthesis, Biomedical and Biotechnological Applications of Carbon and Graphene Quantum Dots. A Review. Environ. Chem. Lett..

[9] Kortshagen U.R., Sankaran R.M., Pereira R.N., Girshick S.L., Wu J.J., Aydil E.S. (2016). Nonthermal Plasma Synthesis of Nanocrystals: Fundamental Principles, Materials, and Applications. Chem. Rev..

[10] Mandracci P., Rivolo P. (2023). Recent Advances in the Plasma-Assisted Synthesis of Silicon-Based Thin Films and Nanostructures. Coatings.

[11] Mangolini L., Thimsen E., Kortshagen U. (2005). High-Yield Plasma Synthesis of Luminescent Silicon Nanocrystals. Nano Lett..

[12] Shen Z., Kim T., Kortshagen U., McMurry P.H., Campbell S.A. (2003). Formation of Highly Uniform Silicon Nanoparticles in High Density Silane Plasmas. J. Appl. Phys..

[13] Knipping J., Wiggers H., Rellinghaus B., Roth P., Konjhodzic D., Meier C. (2004). Synthesis of High Purity Silicon Nanoparticles in a Low Pressure Microwave Reactor. J. Nanosci. Nanotechnol..

[14] Sankaran R.M., Holunga D., Flagan R.C., Giapis K.P. (2005). Synthesis of Blue Luminescent Si Nanoparticles Using Atmospheric-Pressure Microdischarges. Nano Lett..

[15] Cavarroc M., Mikikian M., Tessier Y., Boufendi L. (2008). Instabilities during the Growth of Dust Successive Generations in Silane-Based Plasmas. Phys. Plasmas.

[16] Kim B.-H., Cho C.-H., Kim T.-W., Park N.-M., Sung G.Y., Park S.-J. (2005). Photoluminescence of Silicon Quantum Dots in Silicon Nitride Grown by NH3 and SiH4. Appl. Phys. Lett..

[17] Wei Y., Yu H., Li H., Ming H., Pan K., Huang H., Liu Y., Kang Z. (2013). Liquid-Phase Plasma Synthesis of Silicon Quantum Dots Embedded in Carbon Matrix for Lithium Battery Anodes. Mater. Res. Bull..

[18] Thai V.-P., Tran D.N., Kosugi K., Takahashi K., Sasaki T., Kikuchi T. (2024). One-Step Synthesis of N-Doped Graphene Quantum Dots via Plasma Contacting Liquid for Multiple Heavy Metal Ion Detection. ACS Appl. Nano Mater..

[19] Lanham S.J., Polito J., Shi X., Elvati P., Violi A., Kushner M.J. (2021). Scaling of Silicon Nanoparticle Growth in Low Temperature Flowing Plasmas. J. Appl. Phys..

[20] Lanham S.J., Polito J., Xiong Z., Kortshagen U.R., Kushner M.J. (2022). Pulsed Power to Control Growth of Silicon Nanoparticles in Low Temperature Flowing Plasmas. J. Appl. Phys..

[21] Doğan İ., Kramer N.J., Westermann R.H.J., Dohnalová K., Smets A.H.M., Verheijen M.A., Gregorkiewicz T., van de Sanden M.C.M. (2013). Ultrahigh Throughput Plasma Processing of Free Standing Silicon Nanocrystals with Lognormal Size Distribution. J. Appl. Phys..

[22] Jariwala B.N., Kramer N.J., Petcu M.C., Bobela D.C., van de Sanden M.C.M., Stradins P., Ciobanu C.V., Agarwal S. (2011). Surface Hydride Composition of Plasma-Synthesized Si Nanoparticles. J. Phys. Chem. C.

[23] Sahu B.B., Yin Y., Gauter S., Han J.G., Kersten H. (2016). Plasma Engineering of Silicon Quantum Dots and Their Properties through Energy Deposition and Chemistry. Phys. Chem. Chem. Phys..

[24] Loh K.Q., Andaraarachchi H.P., Ferry V.E., Kortshagen U.R. (2023). Photoluminescent Si/SiO _2_ Core/Shell Quantum Dots Prepared by High-Pressure Water Vapor Annealing for Solar Concentrators, Light-Emitting Devices, and Bioimaging. ACS Appl. Nano Mater..

[25] Matějka F., Galář P., Khun J., Dopita M., Michalcová A., Popelář T., Benedikt J., Kůsová K. (2025). Scalable and Bright: Unlocking Functional Silicon Quantum Dots with near-Unity Internal Quantum Yield through Universal Plasma-Driven Engineering. Nanoscale.

[26] Zhou J., Huang J., Chen H., Samanta A., Linnros J., Yang Z., Sychugov I. (2021). Low-Cost Synthesis of Silicon Quantum Dots with Near-Unity Internal Quantum Efficiency. J. Phys. Chem. Lett..

[27] Chahal S., Macairan J.-R., Yousefi N., Tufenkji N., Naccache R. (2021). Green Synthesis of Carbon Dots and Their Applications. RSC Adv..

[28] Atchudan R., Jebakumar Immanuel Edison T.N., Shanmugam M., Perumal S., Somanathan T., Lee Y.R. (2021). Sustainable Synthesis of Carbon Quantum Dots from Banana Peel Waste Using Hydrothermal Process for in Vivo Bioimaging. Phys. E Low-Dimens. Syst. Nanostruct..

[29] Sharma N., Sharma I., Bera M.K. (2022). Microwave-Assisted Green Synthesis of Carbon Quantum Dots Derived from Calotropis Gigantea as a Fluorescent Probe for Bioimaging. J. Fluoresc..

[30] Tawade A., Thakre A., Ravikumar C., Shende D., Wasewar K. (2025). Green Synthesis of Carbon Quantum Dots Derived from Orange Peel for High-Sensitivity PH Biosensing. Hung. J. Ind. Chem..

[31] Yang S., Cai W., Zhang H., Xu X., Zeng H. (2009). Size and Structure Control of Si Nanoparticles by Laser Ablation in Different Liquid Media and Further Centrifugation Classification. J. Phys. Chem. C.

[32] Calabro R.L., Yang D.-S., Kim D.Y. (2018). Liquid-Phase Laser Ablation Synthesis of Graphene Quantum Dots from Carbon Nano-Onions: Comparison with Chemical Oxidation. J. Colloid Interface Sci..

[33] Morales-Sánchez A., Cardona-Castro M.A., Licea-Jiménez L., Palacios-Huerta L., Coyopol A., Pérez-García S.A., Alvarez-Quintana J., Moreno M. (2022). Study of the Effect of Nitric Acid in Electrochemically Synthesized Silicon Nanocrystals: Tunability of Bright and Uniform Photoluminescence. Nanomaterials.

[34] Desmond L.J., Phan A.N., Gentile P. (2021). Critical Overview on the Green Synthesis of Carbon Quantum Dots and Their Application for Cancer Therapy. Environ. Sci. Nano.

[35] Yang S., Sun J., Li X., Zhou W., Wang Z., He P., Ding G., Xie X., Kang Z., Jiang M. (2014). Large-Scale Fabrication of Heavy Doped Carbon Quantum Dots with Tunable-Photoluminescence and Sensitive Fluorescence Detection. J. Mater. Chem. A Mater..

[36] Nekoueian K., Amiri M., Sillanpää M., Marken F., Boukherroub R., Szunerits S. (2019). Carbon-Based Quantum Particles: An Electroanalytical and Biomedical Perspective. Chem. Soc. Rev..

[37] Li Z., Wang L., Li Y., Feng Y., Feng W. (2019). Frontiers in Carbon Dots: Design, Properties and Applications. Mater. Chem. Front..

[38] Popelář T., Galář P., Matějka F., Morselli G., Ceroni P., Kůsová K. (2023). Universal Radiative Lifetimes in the Long-Lived Luminescence of Si Quantum Dots. J. Phys. Chem. C.

[39] Pringle T.A., Hunter K.I., Brumberg A., Anderson K.J., Fagan J.A., Thomas S.A., Petersen R.J., Sefannaser M., Han Y., Brown S.L. (2020). Bright Silicon Nanocrystals from a Liquid Precursor: Quasi-Direct Recombination with High Quantum Yield. ACS Nano.

[40] Ueda H., Saitow K. (2024). Cost-Effective Ultrabright Silicon Quantum Dots and Highly Efficient LEDs from Low-Carbon Hydrogen Silsesquioxane Polymers. ACS Appl. Mater. Interfaces.

[41] Thiessen A.N., Zhang L., Oliynyk A.O., Yu H., O’Connor K.M., Meldrum A., Veinot J.G.C. (2020). A Tale of Seemingly “Identical” Silicon Quantum Dot Families: Structural Insight into Silicon Quantum Dot Photoluminescence. Chem. Mater..

[42] Zhou J., Ma F., Chen K., Zhao W., Yang R., Qiao C., Shen H., Su W.-S., Lu M., Zheng Y. (2023). The Luminescence Mechanism of Ligand-Induced Interface States in Silicon Quantum Dots. Nanoscale Adv..

[43] Zhu S., Song Y., Zhao X., Shao J., Zhang J., Yang B. (2015). The Photoluminescence Mechanism in Carbon Dots (Graphene Quantum Dots, Carbon Nanodots, and Polymer Dots): Current State and Future Perspective. Nano Res..

[44] Mintz K.J., Zhou Y., Leblanc R.M. (2019). Recent Development of Carbon Quantum Dots Regarding Their Optical Properties, Photoluminescence Mechanism, and Core Structure. Nanoscale.

[45] Elkun S., Ghali M., Sharshar T., Mosaad M.M. (2024). Green Synthesis of Fluorescent N-Doped Carbon Quantum Dots from Castor Seeds and Their Applications in Cell Imaging and PH Sensing. Sci. Rep..

[46] Qandeel N.A., El-Masry A.A., Eid M., Moustafa M.A., El-Shaheny R. (2023). Fast One-Pot Microwave-Assisted Green Synthesis of Highly Fluorescent Plant-Inspired S,N-Self-Doped Carbon Quantum Dots as a Sensitive Probe for the Antiviral Drug Nitazoxanide and Hemoglobin. Anal. Chim. Acta.

[47] Kalaiyarasan G., Joseph J., Kumar P. (2020). Phosphorus-Doped Carbon Quantum Dots as Fluorometric Probes for Iron Detection. ACS Omega.

[48] Deng Y., Long Y., Song A., Wang H., Xiang S., Qiu Y., Ge X., Golberg D., Weng Q. (2023). Boron Dopants in Red-Emitting B and N Co-Doped Carbon Quantum Dots Enable Targeted Imaging of Lysosomes. ACS Appl. Mater. Interfaces.

[49] Dworschak M., Kohlmann N., Matějka F., Galář P., Kienle L., Schäfer J., Benedikt J. (2023). Silicon Nanocrystal Synthesis with the Atmospheric Plasma Source HelixJet. Plasma Process. Polym..

[50] Alessi B., Macias-Montero M., Maddi C., Maguire P., Svrcek V., Mariotti D. (2020). Bridging Energy Bands to the Crystalline and Amorphous States of Si QDs. Faraday Discuss..

[51] León-Guillén R., Muñoz-Rosas A.L., Arenas-Alatorre J.A., Alonso-Huitrón J.C., Pérez-Martínez A.L., Rodríguez-Gómez A. (2022). Experimental Study of the Influence of CH _4_ and H _2_ on the Conformation, Chemical Composition, and Luminescence of Silicon Quantum Dots Inlaid in Silicon Carbide Thin Films Grown by Remote Plasma-Enhanced Chemical Vapor Deposition. ACS Omega.

[52] Cheng C.-H., Lin G.-R. (2020). Si-QD Synthesis for Visible Light Emission, Color Conversion, and Optical Switching. Materials.

[53] Hunter K.I., Andaraarachchi H.P., Kortshagen U.R. (2021). Nonthermal Plasma Synthesized Silicon-Silicon Nitride Core–Shell Nanocrystals with Enhanced Photoluminescence. J. Phys. D Appl. Phys..

[54] Chang G.-Y., Kurniawan D., Chang Y.-J., Chiang W.-H. (2022). Microplasma-Enabled Surfaced-Functionalized Silicon Quantum Dots for Label-Free Detection of Dopamine. ACS Omega.

[55] Ono T., Xu Y., Sakata T., Saitow K. (2022). Designing Efficient Si Quantum Dots and LEDs by Quantifying Ligand Effects. ACS Appl. Mater. Interfaces.

[56] Xu Y., Terada S., Xin Y., Ueda H., Saitow K. (2022). Ligand Effects on Photoluminescence and Electroluminescence of Silicon Quantum Dots for Light-Emitting Diodes. ACS Appl. Nano Mater..

[57] Tao S., Zhou C., Kang C., Zhu S., Feng T., Zhang S.-T., Ding Z., Zheng C., Xia C., Yang B. (2022). Confined-Domain Crosslink-Enhanced Emission Effect in Carbonized Polymer Dots. Light Sci. Appl..

[58] Song L., Shi J., Lu J., Lu C. (2015). Structure Observation of Graphene Quantum Dots by Single-Layered Formation in Layered Confinement Space. Chem. Sci..

[59] Rajender G., Giri P.K. (2016). Formation Mechanism of Graphene Quantum Dots and Their Edge State Conversion Probed by Photoluminescence and Raman Spectroscopy. J. Mater. Chem. C Mater..

[60] Zhao P., Zhang Q., Cao J., Qian C., Ye J., Xu S., Zhang Y., Li Y. (2022). Facile and Green Synthesis of Highly Fluorescent Carbon Quantum Dots from Water Hyacinth for the Detection of Ferric Iron and Cellular Imaging. Nanomaterials.

[61] Malavika J.P., Shobana C., Ragupathi M., Kumar P., Lee Y.S., Govarthanan M., Selvan R.K. (2021). A Sustainable Green Synthesis of Functionalized Biocompatible Carbon Quantum Dots from Aloe Barbadensis Miller and Its Multifunctional Applications. Environ. Res..

[62] Praseetha P.K., Litany R.I.J., Alharbi H.M., Khojah A.A., Akash S., Bourhia M., Mengistie A.A., Shazly G.A. (2024). Green Synthesis of Highly Fluorescent Carbon Quantum Dots from Almond Resin for Advanced Theranostics in Biomedical Applications. Sci. Rep..

[63] Rabiee N., Iravani S., Varma R.S. (2022). Biowaste-Derived Carbon Dots: A Perspective on Biomedical Potentials. Molecules.

[64] Petersen R.J., Thomas S.A., Anderson K.J., Pringle T.A., May S., Hobbie E.K. (2022). Silicon-Carbide Nanocrystals from Nonthermal Plasma: Surface Chemistry and Quantum Confinement. J. Phys. Chem. C.

[65] Wang C., Zhou J., Song M., Lu Z., Chen X., Zheng Y., Xia W. (2021). Synthesis of Ultrafine Silicon Carbide Nanoparticles Using Nonthermal Arc Plasma at Atmospheric Pressure. J. Am. Ceram. Soc..

[66] Viera G., Mikikian M., Bertran E., Cabarrocas P.R.I., Boufendi L. (2002). Atomic Structure of the Nanocrystalline Si Particles Appearing in Nanostructured Si Thin Films Produced in Low-Temperature Radiofrequency Plasmas. J. Appl. Phys..

[67] Ostrikov K., Neyts E.C., Meyyappan M. (2013). Plasma Nanoscience: From Nano-Solids in Plasmas to Nano-Plasmas in Solids. Adv. Phys..

[68] Pal A., Sk M.P., Chattopadhyay A. (2020). Recent Advances in Crystalline Carbon Dots for Superior Application Potential. Mater. Adv..

[69] Bouchoule A., Boufendi L. (1993). Particulate Formation and Dusty Plasma Behaviour in Argon-Silane RF Discharge. Plasma Sources Sci. Technol..

[70] Boufendi L., Bouchoule A. (1994). Particle Nucleation and Growth in a Low-Pressure Argon-Silane Discharge. Plasma Sources Sci. Technol..

[71] Boufendi L., Gaudin J., Huet S., Viera G., Dudemaine M. (2001). Detection of Particles of Less than 5 Nm in Diameter Formed in an Argon–Silane Capacitively Coupled Radio-Frequency Discharge. Appl. Phys. Lett..

[72] Boufendi L., Jouanny M.C., Kovacevic E., Berndt J., Mikikian M. (2011). Dusty Plasma for Nanotechnology. J. Phys. D Appl. Phys..

[73] Ouaras K., Lombardi G., Hassouni K. (2024). Nanoparticles Synthesis in Microwave Plasmas: Peculiarities and Comprehensive Insight. Sci. Rep..

[74] Orazbayev S., Yerlanuly Y., Utegenov A., Moldabekov Z., Gabdullin M., Ramazanov T. (2021). Plasma with Carbon Nanoparticles: Advances and Application. Nanotechnology.

[75] Jurbergs D., Rogojina E., Mangolini L., Kortshagen U. (2006). Silicon Nanocrystals with Ensemble Quantum Yields Exceeding 60%. Appl. Phys. Lett..

[76] Li Z., Kortshagen U.R. (2019). Aerosol-Phase Synthesis and Processing of Luminescent Silicon Nanocrystals. Chem. Mater..

[77] Sinelnikov R., Dasog M., Beamish J., Meldrum A., Veinot J.G.C. (2017). Revisiting an Ongoing Debate: What Role Do Surface Groups Play in Silicon Nanocrystal Photoluminescence?. ACS Photonics.

[78] Müller M., Galář P., Stuchlík J., Kočka J., Kupka J., Kůsová K. (2020). Synthesis and Surface Modification of Light Emitting Silicon Nanoparticles Using Non-Thermal Plasma Techniques. Eur. Phys. J. Appl. Phys..

[79] Vach H., Brulin Q. (2005). Controlled Growth of Silicon Nanocrystals in a Plasma Reactor. Phys. Rev. Lett..

[80] Vach H., Brulin Q., Chaâbane N., Novikova T., Roca i Cabarrocas P., Kalache B., Hassouni K., Botti S., Reining L. (2006). Growth Dynamics of Hydrogenated Silicon Nanoparticles under Realistic Conditions of a Plasma Reactor. Comput. Mater. Sci..

[81] Vach H. (2011). Ultrastable Silicon Nanocrystals Due to Electron Delocalization. Nano Lett..

[82] Jardali F., Keary B.P., Perrotin T., Silva F., Vanel J.-C., Bonnassieux Y., Mazouffre S., Ruth A.A., Leulmi M.E., Vach H. (2021). Hydrogenated Silicon Nanoclusters with a Permanent Electric Dipole Moment for the Controlled Assembly of Silicon-Based Nanostructures. ACS Appl. Nano Mater..

[83] Jardali F., Tran J., Liège F., Florea I., Leulmi M.E., Vach H. (2023). Electric Field-Induced Nano-Assembly Formation: First Evidence of Silicon Superclusters with a Giant Permanent Dipole Moment. Nanomaterials.

[84] Ning N., Vach H. (2010). Deposition Dynamics of Hydrogenated Silicon Clusters on a Crystalline Silicon Substrate under Typical Plasma Conditions. J. Phys. Chem. A.

[85] Le H.-L.T., Jardali F., Vach H. (2018). Deposition of Hydrogenated Silicon Clusters for Efficient Epitaxial Growth. Phys. Chem. Chem. Phys..

[86] Vach H. (2014). Terahertz and Gigahertz Emission from an All-Silicon Nanocrystal. Phys. Rev. Lett..

[87] Ma Y., Wang R., Qin X., Zhang Q., Zhong X. (2023). Plasma Electrochemical Synthesis of Silicon Quantum Dots. J. Phys. D Appl. Phys..

[88] Lee Y., Kim M., Yoo J., Werner J.H., Kortshagen U. (2024). Single-Step Nonthermal Plasma Synthesis of Water-Soluble and Near-Infrared-Emitting Si Quantum Dots for Bioimaging Applications. ACS Appl. Nano Mater..

[89] Kurniawan D., Caielli F., Thyagajaran K., Ostrikov K., Chiang W.H., Pai D.Z. (2024). Operando Time and Space-Resolved Liquid-Phase Diagnostics Reveal the Plasma Selective Synthesis of Nanographenes. Nanoscale.

[90] Yang J.-S., Chang Y.-C., Huang Q.-H., Lai Y.-Y., Chiang W.-H. (2020). Microplasma-Enabled Nanocarbon Assembly for the Diameter-Selective Synthesis of Colloidal Graphene Quantum Dots. Chem. Commun..

[91] Carolan D., Rocks C., Padmanaban D.B., Maguire P., Svrcek V., Mariotti D. (2017). Environmentally Friendly Nitrogen-Doped Carbon Quantum Dots for next Generation Solar Cells. Sustain. Energy Fuels.

[92] Treepet S., Chokradjaroen C., Kim K., Saito N., Watthanaphanit A. (2022). Saccharide-Originated Fluorescent Carbon Dots Synthesized by in-Liquid Plasma with Controlled Orderliness of Carbon Core through Precursor Alteration for Selective and Rapid Metal Ion Detection. Mater. Today Chem..

[93] Pho Q.H., Lin L., Rebrov E.V., Mohsen Sarafraz M., Tran T.T., Tran N.N., Losic D., Hessel V. (2023). Process Intensification for Gram-Scale Synthesis of N-Doped Carbon Quantum Dots Immersing a Microplasma Jet in a Gas-Liquid Reactor. Chem. Eng. J..

[94] Morozova S., Alikina M., Vinogradov A., Pagliaro M. (2020). Silicon Quantum Dots: Synthesis, Encapsulation, and Application in Light-Emitting Diodes. Front. Chem..

[95] Mirabella S., Agosta R., Franzò G., Crupi I., Miritello M., Lo Savio R., Di Stefano M.A., Di Marco S., Simone F., Terrasi A. (2009). Light Absorption in Silicon Quantum Dots Embedded in Silica. J. Appl. Phys..

[96] Thangaraj B., Solomon P.R., Chuangchote S., Wongyao N., Surareungchai W. (2021). Biomass-derived Carbon Quantum Dots—A Review. Part 1: Preparation and Characterization. ChemBioEng Rev..

[97] Wilbrink J.L., Huang C.-C., Dohnalova K., Paulusse J.M.J. (2020). Critical Assessment of Wet-Chemical Oxidation Synthesis of Silicon Quantum Dots. Faraday Discuss..

[98] Hutton G.A.M., Martindale B.C.M., Reisner E. (2017). Carbon Dots as Photosensitisers for Solar-Driven Catalysis. Chem. Soc. Rev..

[99] Li M., Chen T., Gooding J.J., Liu J. (2019). Review of Carbon and Graphene Quantum Dots for Sensing. ACS Sens..

[100] Bian Z., Gomez E., Gruebele M., Levine B.G., Link S., Mehmood A., Nie S. (2025). Bottom-up Carbon Dots: Purification, Single-Particle Dynamics, and Electronic Structure. Chem. Sci..

[101] Cheng X., Lowe S.B., Reece P.J., Gooding J.J. (2014). Colloidal Silicon Quantum Dots: From Preparation to the Modification of Self-Assembled Monolayers (SAMs) for Bio-Applications. Chem. Soc. Rev..

[102] Beri D. (2023). Silicon Quantum Dots: Surface Matter, What Next?. Mater. Adv..

[103] Zhang D., Zhang F., Liao Y., Wang F., Liu H. (2022). Carbon Quantum Dots from Pomelo Peel as Fluorescence Probes for “Turn-Off–On” High-Sensitivity Detection of Fe^3+^ and L-Cysteine. Molecules.

[104] Swathilakshmi, Anandhan S. (2022). Recent Development in Carbon Dot-Based Gas Sensors. Sens. Diagn..

[105] Sahana S., Gautam A., Singh R., Chandel S. (2023). A Recent Update on Development, Synthesis Methods, Properties and Application of Natural Products Derived Carbon Dots. Nat. Prod. Bioprospect..

[106] Glasnov T.N., Kappe C.O. (2011). The Microwave-to-Flow Paradigm: Translating High-Temperature Batch Microwave Chemistry to Scalable Continuous-Flow Processes. Chem.—Eur. J..

[107] Kang C., Huang Y., Yang H., Yan X.F., Chen Z.P. (2020). A Review of Carbon Dots Produced from Biomass Wastes. Nanomaterials.

[108] Nguyen K.G., Baragau I.-A., Gromicova R., Nicolaev A., Thomson S.A.J., Rennie A., Power N.P., Sajjad M.T., Kellici S. (2022). Investigating the Effect of N-Doping on Carbon Quantum Dots Structure, Optical Properties and Metal Ion Screening. Sci. Rep..

[109] Chen W., Shen J., Wang Z., Liu X., Xu Y., Zhao H., Astruc D. (2021). Turning Waste into Wealth: Facile and Green Synthesis of Carbon Nanodots from Pollutants and Applications to Bioimaging. Chem. Sci..

[110] Intartaglia R., Barchanski A., Bagga K., Genovese A., Das G., Wagener P., Di Fabrizio E., Diaspro A., Brandi F., Barcikowski S. (2012). Bioconjugated Silicon Quantum Dots from One-Step Green Synthesis. Nanoscale.

[111] McMahon-Puce W., Mu H., Juodkazis S., Moss D.J., Chon J.W.M. (2025). Optimal Laser Fluence for Pulsed Laser Ablation Synthesis of Silicon Nanoparticles in Liquid. Opt. Express.

[112] Terada S., Xin Y., Saitow K. (2020). Cost-Effective Synthesis of Silicon Quantum Dots. Chem. Mater..

[113] Özbilgin İ.N.G., Yamazaki T., Watanabe J., Sun H.-T., Hanagata N., Shirahata N. (2022). Water-Soluble Silicon Quantum Dots toward Fluorescence-Guided Photothermal Nanotherapy. Langmuir.

[114] Ullal N., Mehta R., Sunil D. (2024). Separation and Purification of Fluorescent Carbon Dots—An Unmet Challenge. Analyst.

[115] Zhi B., Yao X., Wu M., Mensch A., Cui Y., Deng J., Duchimaza-Heredia J.J., Trerayapiwat K.J., Niehaus T., Nishimoto Y. (2021). Multicolor Polymeric Carbon Dots: Synthesis, Separation and Polyamide-Supported Molecular Fluorescence. Chem. Sci..

[116] Han X., Xia C., Wu H., Xie Y., Li R., Sui B., Yu Y., Wang B., Yang B. (2025). Unveiling the Photoluminescence Mechanism of Carbonized Polymer Dots: Evolution and Synergistic Photoluminescence of Multiple Molecular Fluorophores. Angew. Chem. Int. Ed..

[117] Barbagiovanni E.G., Lockwood D.J., Simpson P.J., Goncharova L.V. (2014). Quantum Confinement in Si and Ge Nanostructures: Theory and Experiment. Appl. Phys. Rev..

[118] Kang Z., Liu Y., Lee S.-T. (2011). Small-Sized Silicon Nanoparticles: New Nanolights and Nanocatalysts. Nanoscale.

[119] Brus L.E. (1984). Electron–Electron and Electron-Hole Interactions in Small Semiconductor Crystallites: The Size Dependence of the Lowest Excited Electronic State. J. Chem. Phys..

[120] Huang C.-C., Tang Y., van der Laan M., van de Groep J., Koenderink A.F., Dohnalova K. (2021). Band-Gap Tunability in Partially Amorphous Silicon Nanoparticles Using Single-Dot Correlative Microscopy. ACS Appl. Nano Mater..

[121] Dohnalova K., Hapala P., Kusova K., Infante I. (2020). Electronic Structure Engineering Achieved via Organic Ligands in Silicon Nanocrystals. Chem. Mater..

[122] Poddubny A.N., Dohnalová K. (2014). Direct Band Gap Silicon Quantum Dots Achieved via Electronegative Capping. Phys. Rev. B.

[123] Dohnalová K., Gregorkiewicz T., Kůsová K. (2014). Silicon Quantum Dots: Surface Matters. J. Phys. Condens. Matter.

[124] Ghosh B., Shirahata N. (2020). Influence of Oxidation on Temperature-Dependent Photoluminescence Properties of Hydrogen-Terminated Silicon Nanocrystals. Crystals.

[125] Jariwala B.N., Dewey O.S., Stradins P., Ciobanu C.V., Agarwal S. (2011). In Situ Gas-Phase Hydrosilylation of Plasma-Synthesized Silicon Nanocrystals. ACS Appl. Mater. Interfaces.

[126] Weeks S.L., Macco B., van de Sanden M.C.M., Agarwal S. (2012). Gas-Phase Hydrosilylation of Plasma-Synthesized Silicon Nanocrystals with Short- and Long-Chain Alkynes. Langmuir.

[127] Yu Y., Hessel C.M., Bogart T.D., Panthani M.G., Rasch M.R., Korgel B.A. (2013). Room Temperature Hydrosilylation of Silicon Nanocrystals with Bifunctional Terminal Alkenes. Langmuir.

[128] Chinnathambi S., Chen S., Ganesan S., Hanagata N. (2014). Silicon Quantum Dots for Biological Applications. Adv. Healthc. Mater..

[129] Kumar P., Dua S., Kaur R., Kumar M., Bhatt G. (2022). A Review on Advancements in Carbon Quantum Dots and Their Application in Photovoltaics. RSC Adv..

[130] Wang L., Weng S., Su S., Wang W. (2023). Progress on the Luminescence Mechanism and Application of Carbon Quantum Dots Based on Biomass Synthesis. RSC Adv..

[131] Kwon W., Lim J., Lee J., Park T., Rhee S.-W. (2013). Sulfur-Incorporated Carbon Quantum Dots with a Strong Long-Wavelength Absorption Band. J. Mater. Chem. C Mater..

[132] Bourlinos A.B., Bakandritsos A., Kouloumpis A., Gournis D., Krysmann M., Giannelis E.P., Polakova K., Safarova K., Hola K., Zboril R. (2012). Gd(Iii)-Doped Carbon Dots as a Dual Fluorescent-MRI Probe. J. Mater. Chem..

[133] Liao H., Wang Z., Chen S., Wu H., Ma X., Tan M. (2015). One-Pot Synthesis of Gadolinium(III) Doped Carbon Dots for Fluorescence/Magnetic Resonance Bimodal Imaging. RSC Adv..

[134] Kairdolf B.A., Smith A.M., Stokes T.H., Wang M.D., Young A.N., Nie S. (2013). Semiconductor Quantum Dots for Bioimaging and Biodiagnostic Applications. Annu. Rev. Anal. Chem..

[135] Zhao Y., Su M., Wu Z., Yang W., Du Y., Pang Y., Li N., Li Y., Xing B., Zhang J. (2024). Review of Second Near-Infrared Biosensing, Bioimaging, and Imaging-Guided Therapy with Quantum Dots. ACS Appl. Nano Mater..

[136] Tu C.-C., Awasthi K., Chen K.-P., Lin C.-H., Hamada M., Ohta N., Li Y.-K. (2017). Time-Gated Imaging on Live Cancer Cells Using Silicon Quantum Dot Nanoparticles with Long-Lived Fluorescence. ACS Photonics.

[137] Pan Y., Yang J., Fang Y., Zheng J., Song R., Yi C. (2017). One-Pot Synthesis of Gadolinium-Doped Carbon Quantum Dots for High-Performance Multimodal Bioimaging. J. Mater. Chem. B.

[138] Yuan T., Yuan F., Sui L., Zhang Y., Li Y., Li X., Tan Z., Fan L. (2023). Carbon Quantum Dots with Near-Unity Quantum Yield Bandgap Emission for Electroluminescent Light-Emitting Diodes. Angew. Chem. Int. Ed..

[139] Li J., Zhao H., Zhao X., Gong X. (2023). Boosting Efficiency of Luminescent Solar Concentrators Using Ultra-Bright Carbon Dots with Large Stokes Shift. Nanoscale Horiz..

[140] Zeng S., Chen J., Sun H., Tan W., Liu J., Cao Z. (2026). Time-Resolved Spectroscopy Insights into Charge Transfer Dynamics in Quantum Dot-Based Photocatalytic Hydrogen Evolution. Phys. Chem. Chem. Phys..

[141] Wang J., Jiang J., Li F., Zou J., Xiang K., Wang H., Li Y., Li X. (2023). Emerging Carbon-Based Quantum Dots for Sustainable Photocatalysis. Green Chem..

[142] Kim S., Taehui O., Park C. (2026). Engineering Quantum Dot-Enabled Nanofiltration Membranes for Advanced Ion Separation. Chem. Eng. J..

[143] Mathew S.A., Praveena P., Dhanavel S., Manikandan R., Senthilkumar S., Stephen A. (2020). Luminescent Chitosan/Carbon Dots as an Effective Nano-Drug Carrier for Neurodegenerative Diseases. RSC Adv..

[144] Zhou Q., Gong N., Zhang D., Li J., Han X., Dou J., Huang J., Zhu K., Liang P., Liang X.-J. (2021). Mannose-Derived Carbon Dots Amplify Microwave Ablation-Induced Antitumor Immune Responses by Capturing and Transferring “Danger Signals” to Dendritic Cells. ACS Nano.

[145] Murali G., Kwon B., Kang H., Modigunta J.K.R., Park S., Lee S., Lee H., Park Y.H., Kim J., Park S.Y. (2022). Hematoporphyrin Photosensitizer-Linked Carbon Quantum Dots for Photodynamic Therapy of Cancer Cells. ACS Appl. Nano Mater..

[146] Jiang K., Wang Y., Cai C., Lin H. (2018). Conversion of Carbon Dots from Fluorescence to Ultralong Room-Temperature Phosphorescence by Heating for Security Applications. Adv. Mater..

[147] Baragau I.-A., Power N.P., Morgan D.J., Lobo R.A., Roberts C.S., Titirici M.-M., Middelkoop V., Diaz A., Dunn S., Kellici S. (2021). Efficient Continuous Hydrothermal Flow Synthesis of Carbon Quantum Dots from a Targeted Biomass Precursor for On–Off Metal Ions Nanosensing. ACS Sustain. Chem. Eng..

[148] Supajaruwong S., Porahong S., Wibowo A., Yu Y.-S., Khan M.J., Pongchaikul P., Posoknistakul P., Laosiripojana N., Wu K.C.-W., Sakdaronnarong C. (2023). Scaling-up of Carbon Dots Hydrothermal Synthesis from Sugars in a Continuous Flow Microreactor System for Biomedical Application as in Vitro Antimicrobial Drug Nanocarrier. Sci. Technol. Adv. Mater..

[149] Li Z., Mahajan A., Andaraarachchi H.P., Lee Y., Kortshagen U.R. (2022). Water-Soluble Luminescent Silicon Nanocrystals by Plasma-Induced Acrylic Acid Grafting and PEGylation. ACS Appl. Bio Mater..

[150] Yang Q., Wei L., Zheng X., Xiao L. (2015). Single Particle Dynamic Imaging and Fe^3+^ Sensing with Bright Carbon Dots Derived from Bovine Serum Albumin Proteins. Sci. Rep..

[151] Algar W.R., Massey M., Rees K., Higgins R., Krause K.D., Darwish G.H., Peveler W.J., Xiao Z., Tsai H.-Y., Gupta R. (2021). Photoluminescent Nanoparticles for Chemical and Biological Analysis and Imaging. Chem. Rev..

[152] Han B., Hu X., Zhang X., Huang X., An M., Chen X., Zhao D., Li J. (2022). The Fluorescence Mechanism of Carbon Dots Based on the Separation and Identification of Small Molecular Fluorophores. RSC Adv..

[153] Watanabe J., Yamada H., Sun H.-T., Moronaga T., Ishii Y., Shirahata N. (2021). Silicon Quantum Dots for Light-Emitting Diodes Extending to the NIR-II Window. ACS Appl. Nano Mater..

[154] Sharma A., Raut S.S., Shukla A., Singh A., Mishra A. (2026). The Chemical Record of Carbon Quantum Dots: Charting the Course From Mechanistic Insights to Sustainable Technologies. Chem. Rec..

